# Global, regional, and national burden of gastric cancer attributable to diet high in sodium from 1990 to 2021

**DOI:** 10.3389/fnut.2025.1674979

**Published:** 2025-10-07

**Authors:** Chao Ma, Bing Yan, Ge Li, Yongsheng Jiang

**Affiliations:** Department of Gastrointestinal Surgery, Central Hospital Affiliated to Shandong First Medical University, Jinan Central Hospital, Jinan, China

**Keywords:** diet high in sodium, gastric cancer, DALYs, SDI, cross-national inequality

## Abstract

**Background:**

This study, based on the Global Burden of Disease (GBD) 2021 database, investigates the global and regional burden of gastric cancer attributable to diet high in sodium (GC-DHIS) from 1990 to 2021 and projects trends through 2045, with the aim of providing evidence to inform public health policymaking.

**Methods:**

Data were obtained from GBD 2021, covering 204 countries and territories. Disability-adjusted life years (DALYs) were used as the primary metric. The Das Gupta method was applied to decompose the drivers of changes in disease burden, and the Nordpred model was used to forecast future trends.

**Results:**

Between 1990 and 2021, the global number of DALYs attributable to GC-DHIS decreased by 2%, while deaths increased by 12%. The largest absolute number of DALYs was observed in middle SDI regions, whereas the most rapid growth occurred in low-middle SDI regions. Health inequality analysis revealed that the burden of GC-DHIS was disproportionately concentrated in regions with higher levels of social development. Projections from 2022 to 2045 suggest a significant increase in DALYs and deaths, with a faster rise among males. However, age-standardized DALY rates (ASDR) and age-standardized mortality rates (ASMR) are expected to decline substantially.

**Conclusion:**

Despite the global decline in ASDR and ASMR of GC-DHIS, the absolute burden is expected to rise in specific regions and populations, highlighting the need for targeted prevention and control strategies.

## Introduction

Gastric cancer (GC) is one of the most common malignant tumors worldwide, characterized by high incidence and mortality rates (1, 2). Globally, it ranks as the fifth most prevalent cancer, with particularly high incidence in East Asia (3). The pathogenesis of GC is multifactorial. *Helicobacter pylori* infection is a major etiological factor and is strongly associated with precancerous conditions such as chronic atrophic gastritis, intestinal metaplasia, and dysplasia (4). In addition, dietary factors—including excessive salt intake, frequent consumption of pickled foods, and insufficient intake of fresh fruits and vegetables—are closely linked to an increased risk of GC (5, 6).

Substantial evidence indicates that a high-sodium diet significantly elevates GC risk (7, 8). Excess salt can directly damage the gastric mucosa, stimulate cellular proliferation, and induce endogenous mutations (9). Furthermore, nitrates and nitrites present in high-salt foods may generate carcinogenic N-nitroso compounds. High sodium intake can also intensify *H. pylori* infection by enhancing its carcinogenic potential, thereby promoting chronic inflammation and the progression of precancerous lesions (10, 11). Epidemiological studies have shown that a high-salt diet increases the risk of GC by 55%, and excessive sodium intake is estimated to account for approximately 16.6% of GC cases worldwide (12).

To provide a more comprehensive understanding of gastric cancer attributable to a diet high in sodium (GC-DHIS), we conducted a systematic analysis based on the Global Burden of Disease 2021 (GBD 2021) database. This study examines the global, regional, and national epidemiological trends of GC-DHIS. By precisely analyzing the dynamic changes in disease burden over time, we were able to identify regions with the most significant increases during specific periods, thereby offering strong and targeted evidence to support the development of cancer prevention and control strategies. In addition, we explore cross-national health inequalities and apply the Nordpred model to project GC-DHIS trends from 2022 to 2045. The findings of this study are expected to provide essential evidence for public health policymaking and serve as an important reference for global GC prevention efforts.

## Methods

### Data sources and disease definition

All data in this study were obtained from the GBD 2021 database. Led by the Institute for Health Metrics and Evaluation (IHME) at the University of Washington, GBD 2021 provides estimates for 204 countries and territories, 811 subnational locations, 371 diseases and injuries, and 88 risk factors from 1990 to 2021 (13–15). The GBD employs a unified modeling framework and standardized methodologies to integrate diverse data sources—including vital registration systems, hospital records, population-based surveys, cohort studies, and systematic reviews—ensuring comparability across regions and over time (16, 17). This study focused on GC (ICD-10 code: C16), which encompasses gastric adenocarcinoma, gastric sarcoma, and other malignant tumors of the stomach (18). All disease definitions and classifications followed GBD standards.

### Disability-adjusted life years

Disability-Adjusted Life Years (DALYs) were used as the primary metric to quantify the overall burden of GC. DALYs are calculated as the sum of Years of Life Lost (YLLs) due to premature mortality and Years Lived with Disability (YLDs) (19). YLLs are derived by multiplying the number of deaths by the standard life expectancy at the age of death. YLDs are estimated based on the number of prevalent cases, corresponding disability weights, and average disease duration.

### Decomposition analysis

To identify the main drivers of change in the GC burden, we applied the Das Gupta standardization decomposition method. This approach attributes variations in DALYs and deaths to three independent components: population growth, population aging, and epidemiological changes (20). By controlling variables and simulating the contribution of each factor separately, this method allows clear differentiation of the underlying drivers of increasing or decreasing disease burden.

### Cross-country inequality analysis

In terms of health inequality analysis, this study introduced two methods—Slope Index of Inequality (SII) and Concentration Index (CI)—to assess disparities in health burden across countries (20, 21). The SII quantifies the absolute difference in health outcomes between the highest and lowest SDI countries using regression modeling. The CI, based on the Lorenz curve, measures the relative distribution of disease burden across countries with different SDI levels. A CI greater than 0 indicates that the burden is concentrated in high-SDI countries, whereas a CI less than 0 suggests concentration in low-SDI countries.

### Prediction analysis

To forecast future trends in GC burden, we used the Nordpred model, which provides medium- and long-term projections (22). Based on an Age–Period–Cohort (APC) regression framework, the model simultaneously accounts for age structure, period effects, and cohort effects, allowing robust prediction of DALY and mortality trends.

## Results

### Global and regional burden of GC-DHIS

**Figure 1:**
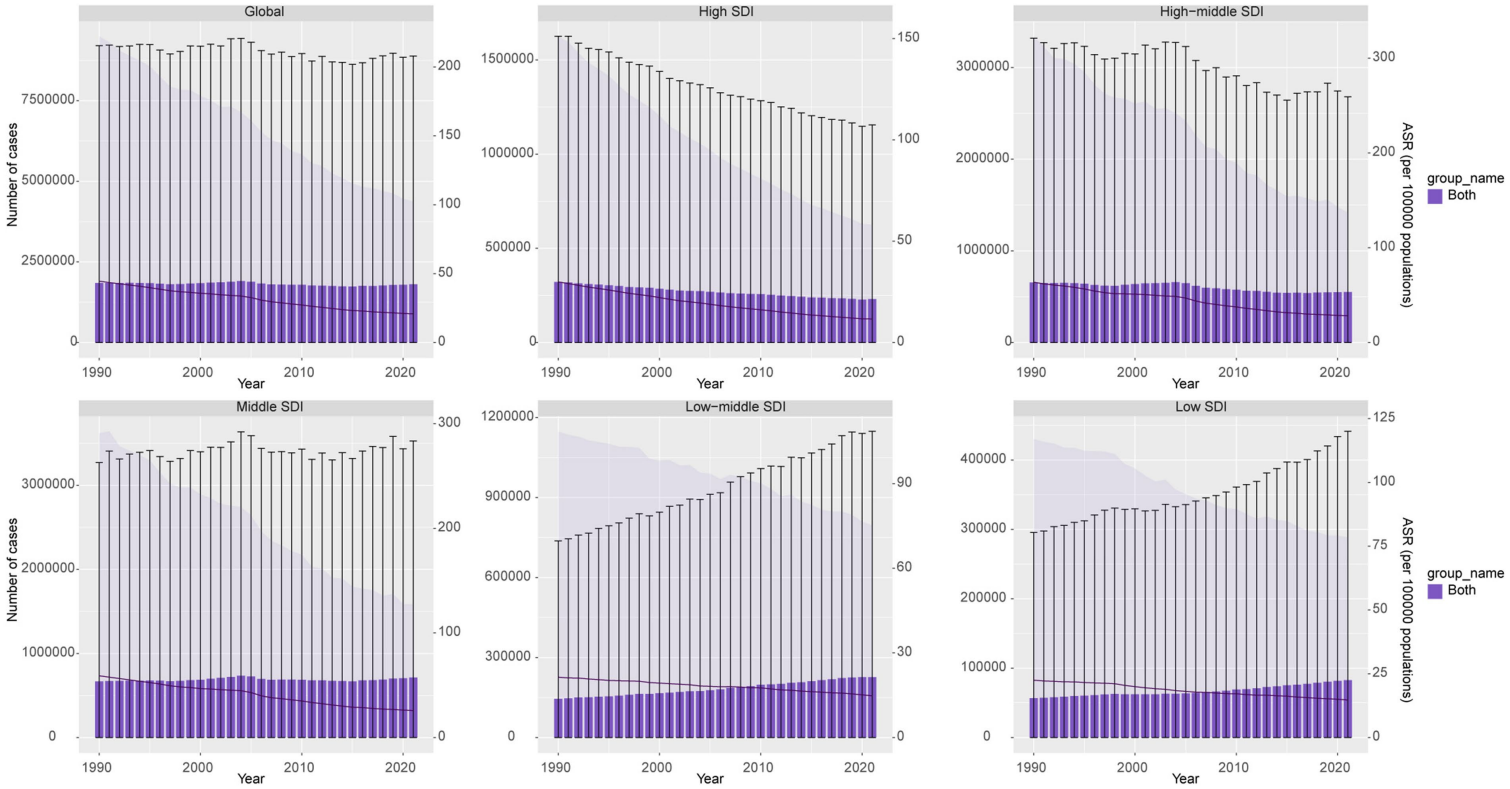
DALYs cases and ASDR of GC-DHIS from 1990 to 2021.

In 2021, the global number of DALYs due to GC-DHIS was 1,804,592, compared to 1,845,617 in 1990, representing a percentage change of −2%. The Middle SDI regions had the highest number of DALYs (712,581.6), with a percentage change of 7%, while the Low-middle SDI regions experienced the fastest increase, with a percentage change of 56%. Globally, the age-standardized DALY rate (ASDR) was 20.78 per 100,000 population, with an estimated annual percentage change (EAPC) of −2.56 (95% CI, −2.64 to −2.47). The High-middle SDI regions had the highest ASDR at 28.14 per 100,000, with an EAPC of −2.76 (95% CI, −2.90 to −2.63). Among the 21 GBD regions, Oceania had the fastest increase in DALYs, with a percentage change of 101%, while East Asia had the highest ASDR (41.09 per 100,000, EAPC, −2.88; 95% CI: −3.06 to −2.70) (Figure 1 and Table 1).

**Table 1:** Disability-adjusted life years (DALYs) and age-standardized DALY rate (ASDR) of GC-DHIS in 1990 and 2021, and the PC and EAPC from 1990 to 2021.

Location	1990_DALYs cases (95% UI)	2021_DALYs cases (95% UI)	Percentage change	1990_ASDR_per 100,000 (95% UI)	2021_ASDR_per 100,000 (95% UI)	EAPC (95% CI)
Andean Latin America	13409.97 (0–68083.61)	23127.69 (0–116683.7)	0.72	62.37 (0–316.56)	38.45 (0–193.86)	−1.79 (−1.93–−1.64)
Australasia	2602.89 (0–14210.09)	2808.9 (0–15482.52)	0.08	11.25 (0–61.37)	5.72 (0–31.14)	−2.16 (−2.26–−2.05)
Caribbean	5804.55 (0–30337.97)	7607.75 (0–40990.47)	0.31	21.93 (0–114.63)	14.24 (0–76.67)	−1.31 (−1.41–−1.21)
Central Asia	28789.8 (0–143224.5)	20435.5 (0–105882.3)	−0.29	57.97 (0–288.43)	23.08 (0–119.44)	−2.8 (−2.88–−2.73)
Central Europe	55098.53 (0–277908.9)	34860.24 (0–170908.9)	−0.37	36.72 (0–185.31)	16.71 (0–81.88)	−2.62 (−2.71−−2.54)
Central Latin America	33796.36 (0–170527.8)	57296.69 (0–297890.1)	0.7	38.56 (0–194.24)	22.41 (0–116.52)	−2 (−2.09–−1.92)
Central Sub-Saharan Africa	4236.6 (0–24980.08)	7863.51 (0–46176.26)	0.86	16.97 (0–100.02)	12.54 (0–73.89)	−1.02 (−1.06–−0.98)
East Asia	910,166 (−0.04–4,421,005)	906420.4 (−0.01–4,574,158)	0	96.58 (0–469.07)	41.09 (0–206.63)	−2.88 (−3.06–−2.7)
Eastern Europe	173732.8 (0–903,204)	79239.65 (0–402533.4)	−0.54	61.63 (0–320.72)	23.54 (0–119.38)	−3.4 (−3.52–−3.28)
Eastern Sub-Saharan Africa	19716.43 (0–99792.75)	24470.02 (0–129514.7)	0.24	23.37 (0–118.12)	12.94 (0–67.73)	−2.21 (−2.32–−2.11)
Global	1,845,617 (−0.03–9,206,158)	1,804,592 (0–8,884,379)	−0.02	44.53 (0–222.31)	20.78 (0–102.38)	−2.56 (−2.64–−2.47)
High-income Asia Pacific	151,938 (0–743669.5)	99016.59 (0–495285.3)	−0.35	74.62 (0–365.66)	22.57 (0–112.13)	−3.92 (−3.97–−3.87)
High-income North America	31635.14 (0–165388.2)	32630.83 (0–166,471)	0.03	9.34 (0–48.84)	5.52 (0–28.1)	−1.73 (−1.77–−1.68)
High-middle SDI	653552.2 (−0.02–3,320,830)	551396.4 (0–2,681,101)	−0.16	63.6 (0–322.96)	28.14 (0–136.92)	−2.76 (−2.9–−2.63)
High SDI	321495.7 (0–1,625,678)	230573.7 (0–1,154,960)	−0.28	29.89 (0–151.09)	11.6 (0–58.09)	−3.11 (−3.13–−3.08)
Low-middle SDI	145052.7 (0–737564.4)	226298.3 (0–1,148,265)	0.56	21.39 (0–108.46)	14.78 (0–75.09)	−1.13 (−1.16–−1.09)
Low SDI	56793.8 (0–295505.2)	82603.09 (0–441280.6)	0.45	22.47 (0–116.97)	14.71 (0–78.41)	−1.41 (−1.47–−1.36)
Middle SDI	667339.3 (−0.02–3,270,306)	712581.6 (0–3,527,047)	0.07	59.02 (0–290.91)	25.82 (0–127.58)	−2.79 (−2.91–−2.68)
North Africa and Middle East	37024.39 (0–214620.6)	54759.4 (0–325675.9)	0.48	19.66 (0–114.77)	11 (0–65.92)	−1.83 (−1.9–−1.77)
Oceania	1064.58 (0–5908.38)	2143.71 (0–11558.1)	1.01	32.71 (0–178.43)	25.52 (0–134.98)	−0.82 (−0.89–−0.76)
South Asia	112571.2 (0–573787.7)	180806.1 (0–908078.1)	0.61	17.02 (0–87.29)	11.45 (0–57.6)	−1.19 (−1.26–−1.11)
Southeast Asia	66675.66 (0–336540.3)	97670.36 (0–497996.7)	0.46	23.49 (0–118.14)	13.98 (0–71.25)	−1.85 (−1.91–−1.78)
Southern Latin America	16277.62 (0–82265.41)	16598.73 (0–82996.48)	0.02	35 (0–176.91)	19.43 (0–97.04)	−1.73 (−1.84–−1.62)
Southern Sub-Saharan Africa	4431.39 (0–23681.44)	7339.7 (0–39115.11)	0.66	14.62 (0–78.11)	11.52 (0–62.18)	−0.75 (−1.08–−0.43)
Tropical Latin America	35939.59 (0–179651.9)	49,223 (0–252502.2)	0.37	37.33 (0–186.56)	18.88 (0–96.83)	−2.33 (−2.39–−2.27)
Western Europe	127536.1 (0–667,811)	76170.59 (0–392854.7)	−0.4	22.85 (0–119.24)	8.93 (0–45.98)	−2.96 (−3.05–−2.88)
Western Sub-Saharan Africa	13169.32 (0–70651.2)	24102.24 (0–126886.4)	0.83	14.02 (0–75.09)	11.32 (0–59.28)	−0.48 (−0.55–−0.4)

In 2021, the global number of deaths due to GC-DHIS was 75,661.15, up from 67,844.54 in 1990, representing a 12% increase. The Middle SDI regions had the highest number of deaths (28,816.28), with a percentage change of 24%, while the Low-middle SDI regions showed the fastest increase, with a percentage change of 69%. Additionally, the global age-standardized mortality rate (ASMR) for GC-DHIS was 0.89 per 100,000 population, with an EAPC of −2.26 (95% CI: −2.35 to −2.18). The High-middle SDI regions recorded the highest ASMR at 1.18 per 100,000, with an EAPC of −2.43 (95% CI: −2.56 to −2.31). Among the 21 GBD regions, Oceania showed the fastest increase in death cases, with a percentage change of 101%. East Asia had the highest ASMR, at 1.76 per 100,000, with an EAPC of −2.54 (95% CI: −2.74 to −2.34) (Supplementary Figure 1 and Table 2).

**Table 2:** Deaths and age-standardized mortality rate (ASMR) of GC-DHIS in 1990 and 2021, and the PC and EAPC from 1990 to 2021.

Location	1990_Death cases (95% UI)	2021_Death cases (95% UI)	Percentage change	1990_ASMR_per 100,000 (95% UI)	2021_ASMR_per 100,000 (95% UI)	EAPC (95% CI)
Andean Latin America	522.25 (0–2644.79)	988.32 (0–4998.75)	0.89	2.67 (0–13.48)	1.71 (0–8.65)	−1.64 (−1.79–−1.5)
Australasia	113.13 (0–630.21)	138.14 (0–787.55)	0.22	0.49 (0–2.7)	0.25 (0–1.41)	−2.1 (−2.22–−1.99)
Caribbean	236.21 (0–1222.59)	311.09 (0–1670.65)	0.32	0.94 (0–4.83)	0.58 (0–3.1)	−1.48 (−1.55–−1.4)
Central Asia	998.02 (0–4972.32)	722.78 (0–3724.68)	−0.28	2.13 (0–10.58)	0.9 (0–4.6)	−2.54 (−2.64–−2.44)
Central Europe	2259.36 (0–11339.28)	1,600 (0–7857.9)	−0.29	1.54 (0–7.69)	0.71 (0–3.49)	−2.58 (−2.67–−2.5)
Central Latin America	1316.45 (0–6624.97)	2295.17 (0–11894.87)	0.74	1.7 (0–8.53)	0.93 (0–4.83)	−2.18 (−2.26–−2.1)
Central Sub-Saharan Africa	141.97 (0–838.2)	262.04 (0–1543.94)	0.85	0.68 (0–4.03)	0.51 (0–2.99)	−0.96 (−1–−0.92)
East Asia	31815.6 (0–155223.57)	37861.8 (0–188112.18)	0.19	3.77 (0–18.42)	1.76 (0–8.69)	−2.54 (−2.74–−2.34)
Eastern Europe	6322.64 (0–32995.76)	3240.56 (0–16644.57)	−0.49	2.25 (0–11.72)	0.92 (0–4.74)	−3.11 (−3.21–−3.01)
Eastern Sub-Saharan Africa	657.5 (0–3325.25)	852.33 (0–4457.26)	0.3	0.9 (0–4.5)	0.54 (0–2.79)	−1.9 (−1.99–−1.81)
Global	67844.54 (0–339512.73)	75661.15 (0–372194.01)	0.12	1.74 (0–8.74)	0.89 (0–4.37)	−2.26 (−2.35–−2.18)
High-income Asia Pacific	6059.35 (0–29936.84)	5863.7 (0–29532.38)	−0.03	3.09 (0–15.26)	1.09 (0–5.43)	−3.44 (−3.48–−3.39)
High-income North America	1413.91 (0–7465.54)	1481.69 (0–7675.1)	0.05	0.4 (0–2.1)	0.23 (0–1.17)	−1.88 (−1.92–−1.83)
High-middle SDI	24047.5 (0–122047.5)	23438.81 (0–114552.7)	−0.03	2.44 (0–12.41)	1.18 (0–5.79)	−2.43 (−2.56–−2.31)
High SDI	13705.92 (0–69402.89)	12208.66 (0–61721.58)	−0.11	1.24 (0–6.27)	0.54 (0–2.73)	−2.72 (−2.75–−2.7)
Low-middle SDI	4880.69 (0–24751.39)	8255.39 (0–41815.52)	0.69	0.82 (0–4.11)	0.59 (0–3)	−0.95 (−1–−0.91)
Low SDI	1904.18 (0–9916.48)	2893.77 (0–15443.34)	0.52	0.86 (0–4.48)	0.6 (0–3.18)	−1.14 (−1.19–−1.08)
Middle SDI	23250.29 (0–115302.5)	28816.28 (0–141923.4)	0.24	2.3 (0–11.48)	1.11 (0–5.43)	−2.47 (−2.6–−2.35)
North Africa and Middle East	1257.34 (0–7340.69)	1976.55 (0–11965.72)	0.57	0.75 (0–4.4)	0.45 (0–2.75)	−1.58 (−1.64–−1.51)
Oceania	35.84 (0–195.58)	72.09 (0–380.84)	1.01	1.36 (0–7.12)	1.06 (0–5.52)	−0.83 (−0.89–−0.78)
South Asia	3630.86 (0–18682.73)	6498.88 (0–32699.54)	0.79	0.62 (0–3.21)	0.45 (0–2.25)	−0.95 (−1.03–−0.86)
Southeast Asia	2263.81 (0–11384.92)	3571.6 (0–18178.29)	0.58	0.91 (0–4.61)	0.56 (0–2.89)	−1.71 (−1.78–−1.65)
Southern Latin America	685.5 (0–3454.82)	751.84 (0–3766.66)	0.1	1.52 (0–7.61)	0.85 (0–4.26)	−1.69 (−1.8–−1.58)
Southern Sub-Saharan Africa	148.19 (0–796.35)	254.56 (0–1387.35)	0.72	0.55 (0–3)	0.45 (0–2.48)	−0.69 (−1.01–−0.37)
Tropical Latin America	1355.85 (0–6802.21)	1988.04 (0–10206.04)	0.47	1.58 (0–7.95)	0.78 (0–4.01)	−2.36 (−2.42–−2.31)
Western Europe	6132.02 (0–32392.14)	4049.66 (0–21330.73)	−0.34	1.04 (0–5.47)	0.41 (0–2.1)	−3 (−3.1–−2.9)
Western Sub-Saharan Africa	478.73 (0–2558.73)	880.31 (0–4609.46)	0.84	0.57 (0–3.06)	0.49 (0–2.54)	−0.28 (−0.37–−0.2)

### National burden of GC-DHIS

**Figure 2:**
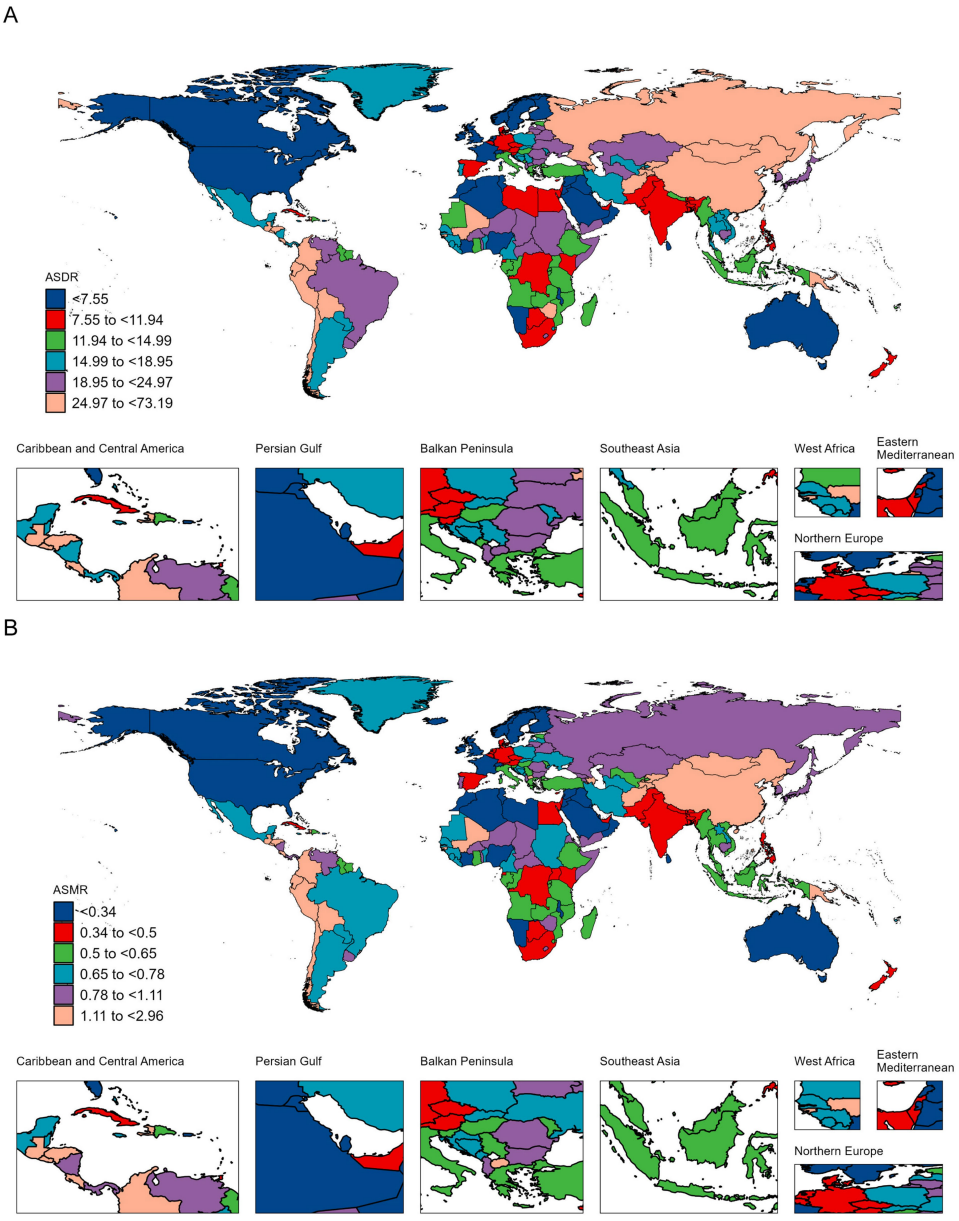
ASDR **(A)** and ASMR **(B)** of GC-DHIS per 100,000 population in 2021, by country.

Over the past 32 years, approximately 65% of countries showed an increasing trend in GC-DHIS-related DALYs. The fastest-growing countries in DALYs were Djibouti (234%), United Arab Emirates (231%), and Honduras (206%). Approximately 70% of countries also experienced an increase in death cases, with the fastest increases observed in Djibouti (254%), Honduras (248%), and United Arab Emirates (231%) (Figure 2A).

The countries with the highest ASDR were Mongolia (73.19 per 100,000), Bolivia (56.47 per 100,000), and the Democratic People’s Republic of Korea (48.47 per 100,000). The highest ASMR were observed in Mongolia (2.96 per 100,000), Bolivia (2.58 per 100,000), and Guatemala (1.98 per 100,000). In terms of trends, the fastest increases in ASDR were recorded in Egypt, Lesotho, and Zimbabwe, with EAPCs of 2.07 (95% CI: 1.52–2.63), 1.76 (95% CI: 1.29–2.24), and 1.38 (95% CI: 0.81–1.95), respectively. The fastest increases in ASMR were also seen in Egypt, Lesotho, and Zimbabwe, with EAPCs of 2.49 (95% CI: 1.88–3.10), 1.49 (95% CI: 1.07–1.92), and 1.11 (95% CI: 0.62–1.59), respectively (Figure 2B and Supplementary Tables 1, 2).

### Age and sex differences in the burden of GC-DHIS

**Figure 3:**
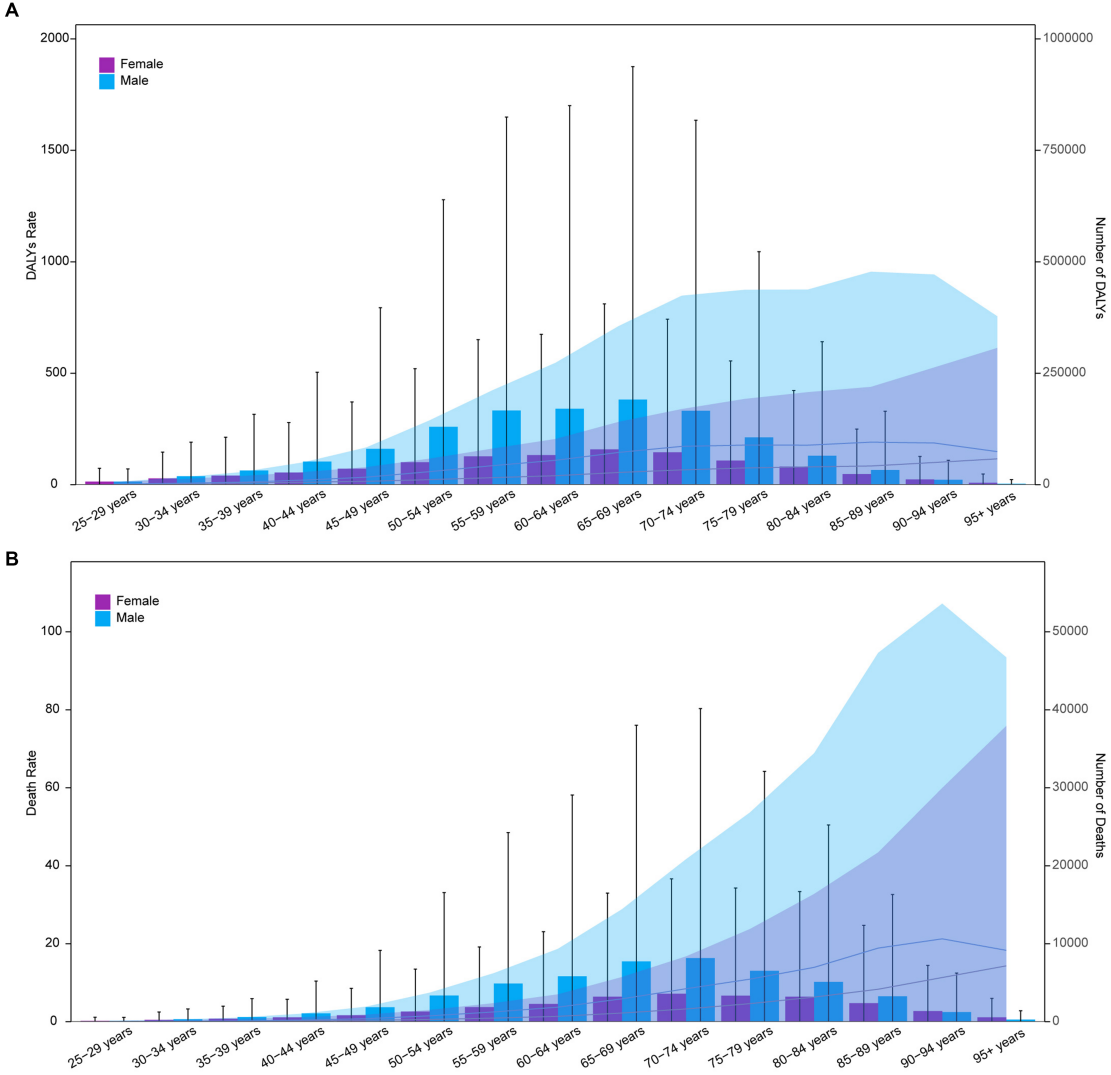
Age-specific numbers and rates of DALYs **(A)** and deaths **(B)** of GC-DHIS by age and sex in 2021.

In 2021, the highest DALYs cases for both sexes occurred in the 65–69 age group, with 190,995 cases for males and 79,182 for females. The highest number of deaths occurred in the 70–74 age group, with 8,159 male and 3,589 female deaths. The highest ASDR among females was observed in the 95 + age group (116 per 100,000), while for males it was in the 85–89 age group (191 per 100,000). The highest ASMR for females was in the 95 + age group (14.3 per 100,000), and for males in the 90–94 age group (21.3 per 100,000) (Figures 3A,B).

### Relationship between the burden of GC-DHIS and SDI

**Figure 4:**
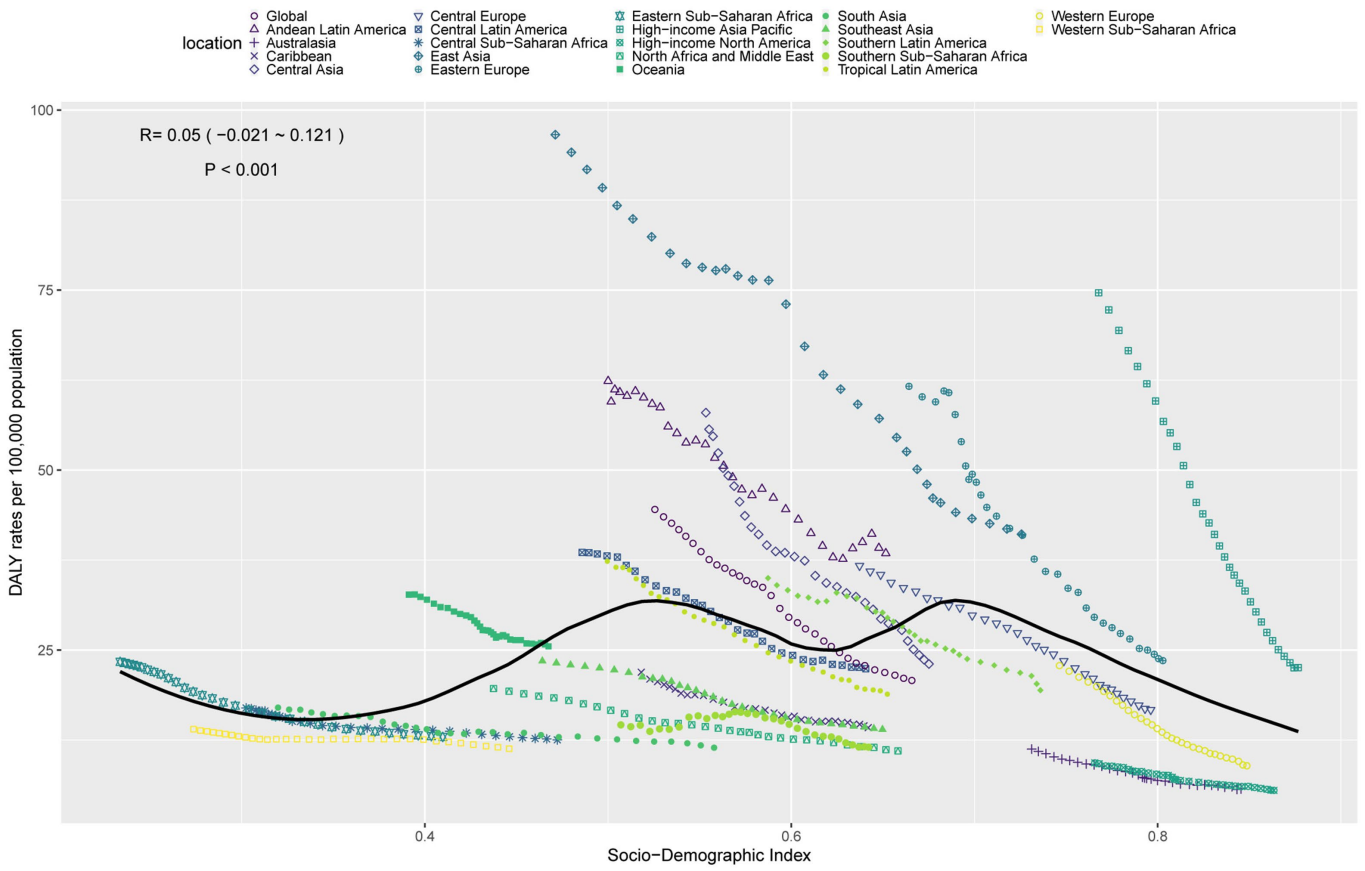
ASDR of GC-DHIS in 21 GBD regions by SDI, 1990–2021.

In 2021, ASDR and ASMR for GC-DHIS showed a positive correlation with SDI (Figure 4 and Supplementary Figure 2). As SDI increased, ASDR and ASMR exhibited a W-shaped pattern. Notably, when the SDI exceeded 0.7, ASDR and ASMR showed a marked decline. Some regions—such as High-income Asia Pacific, Eastern Europe, and East Asia—had a higher disease burden than expected, while others—such as Australasia, Western Europe, and Southern Sub-Saharan Africa—had a lower-than-expected burden.

### Decomposition analysis of the burden of GC-DHIS

**Figure 5:**
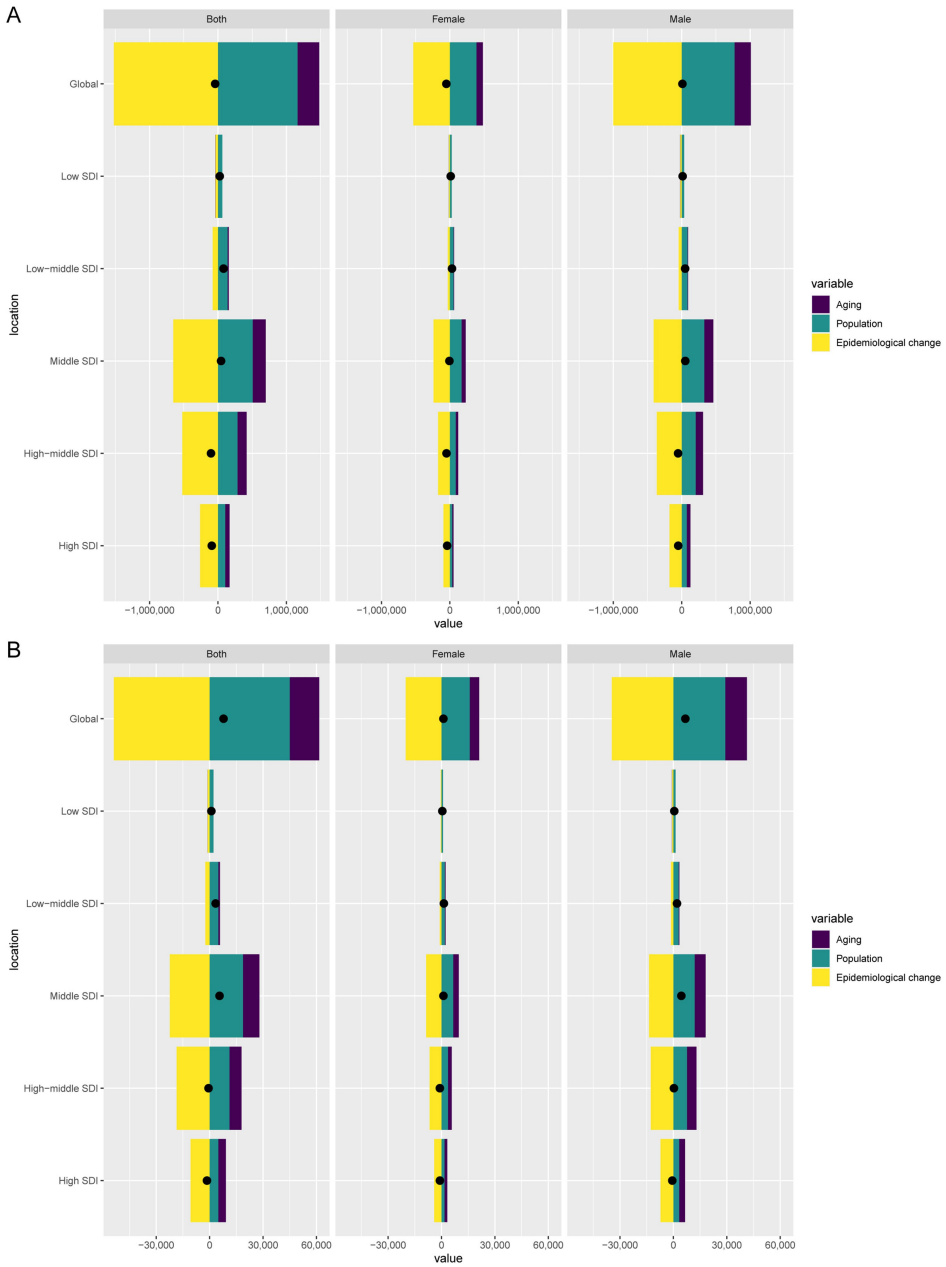
Decomposition analysis of changes in DALYs **(A)** and deaths **(B)** of GC-DHIS between 1990 and 2021 across SDI regions.

Over the past 32 years, global GC-DHIS-related DALYs decreased by 41,025.28. Of this change, aging contributed 316,903.01 (−772.46%), population growth contributed 1,163,570.06 (−2,836.23%), and epidemiological change contributed −1,521,498.36 (3,708.69%). Among males, DALYs increased by 10,231.96, with aging contributing 238,400.95 (2,329.96%), population growth contributing 771,902.63 (7,544.03%), and epidemiological change contributing −1,000,071.62 (−9,774%). Among females, DALYs decreased by 51,257.24, with aging contributing 93,628.57 (−182.66%), population growth 391,667.43 contributing (−764.05%), and epidemiological change contributing −533,576.56 (1,040.98%) (Figure 5A).

Globally, GC-DHIS-related deaths increased by 7,816.61, with aging contributing 16,534.17 (211.53%), population growth contributing 45,023.99 (576%), and epidemiological change contributing −53,741.54 (−687.53%). Among males, deaths increased by 6,731.52, with aging contributing 12,191.44 (181.11%), population growth contributing 29,129.89 (432.74%), and epidemiological change contributing −34,589.82 (−513.85%). Among females, deaths increased by 1,085.09, with aging contributing 5,358.93 (493.87%), population growth contributing 15,821.85 (1,458.11%), and epidemiological change contributing −20,095.69 (−1,851.98%) (Figure 5B).

### Cross-country inequality in the burden of GC-DHIS

**Figure 6:**
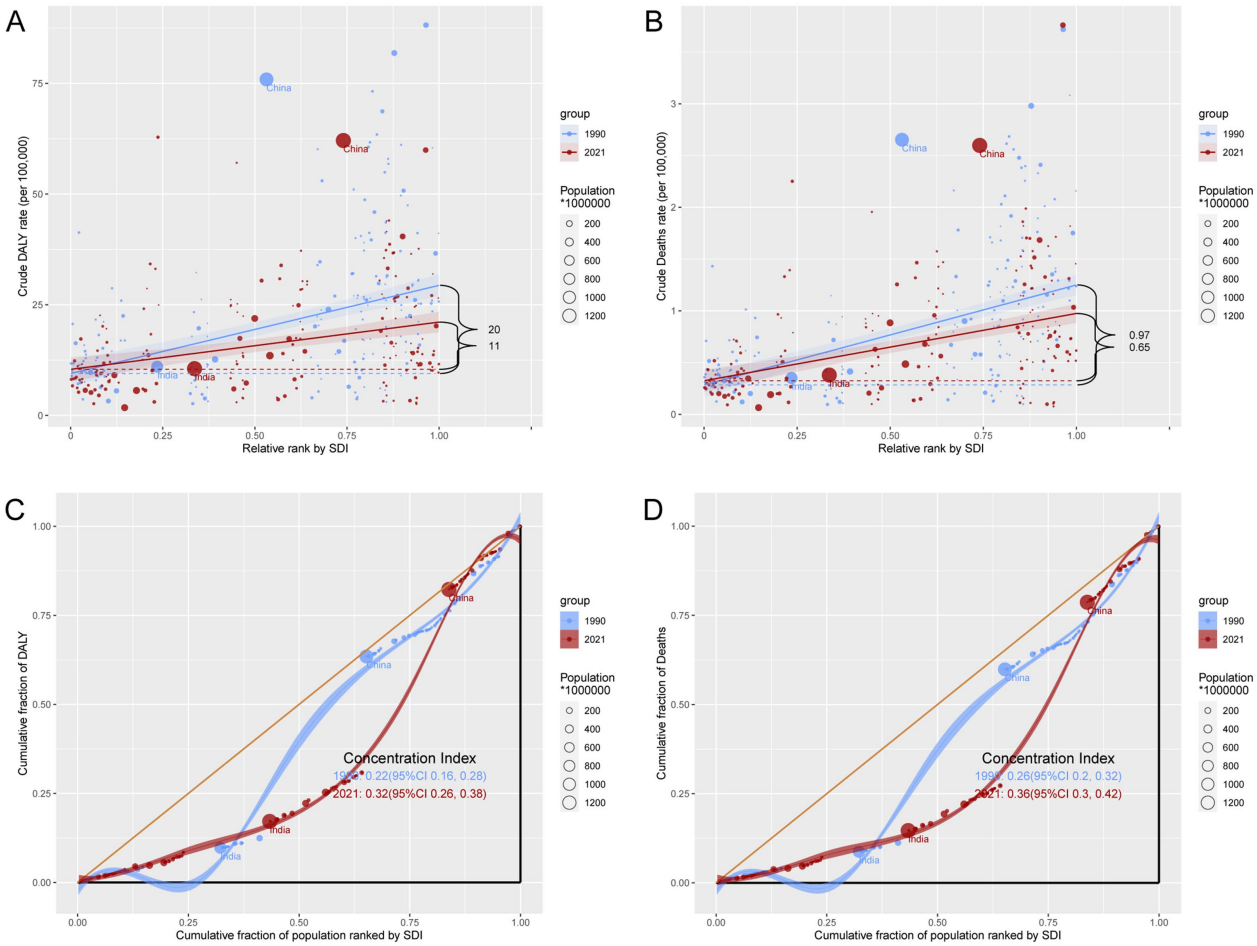
Inequality analysis of DALYs and mortality in GC-DHIS in 1990 and 2021 across the world. **(A)** Health inequality regression curves for DALYs. **(B)** Health inequality regression curves for mortality. **(C)** Concentration curves for DALYs. **(D)** Concentration curves for mortality.

The SII for DALYs rates declined from 20 in 1990 to 11 in 2021, and the CI for DALYs rates dropped from 0.97 to 0.65 over the same period, indicating a narrowing of absolute inequalities between the highest and lowest SDI countries. However, the CI for DALYs rates increased from 0.22 in 1990 to 0.32 in 2021, and for death rates from 0.26 to 0.36, suggesting that the burden has become increasingly concentrated in higher-SDI regions (Figures 6A–D).

### Forecasted burden of GC-DHIS

**Figure 7:**
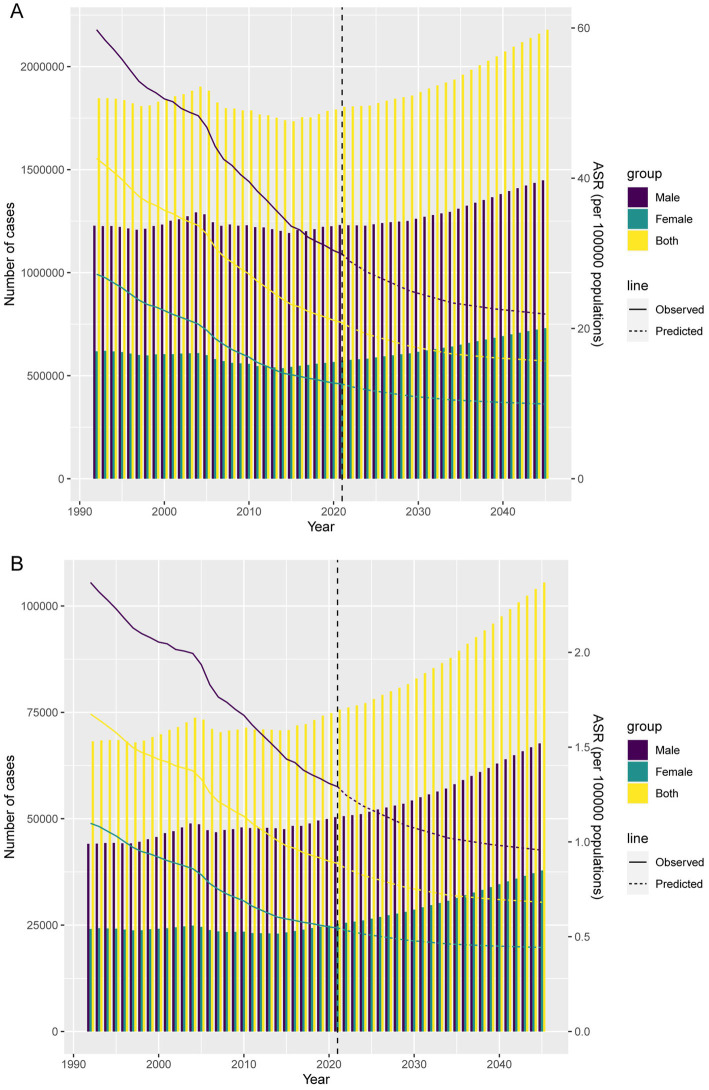
Projections of the temporal trends of the number of DALYs cases, mortality cases, ASDR, and ASMR of GC-DHIS globally up to 2045. **(A)** The number and ASDR of GC-DHIS by year and gender. **(B)** The number and ASMR of GC-DHIS by year and gender.

Forecast analysis indicates a significant upward trend in DALYs and death cases from 2022 to 2045, with male cases consistently higher than female cases (Figures 7A,B). By 2045, the number of DALYs cases is projected to reach 1,447,797 for males and 730,873 for females. The number of death cases is expected to reach 67,686 for males and 37,828 for females. In contrast, the ASDR and ASMR are projected to decline significantly over time. By 2045, the ASDR is expected to be 21.92 per 100,000 for males and 9.95 per 100,000 for females. The ASMR is projected to be 0.96 per 100,000 for males and 0.44 per 100,000 for females.

## Discussion

The pathogenesis of GC is multifactorial, involving *H. pylori* infection, genetic susceptibility, and other dietary habits such as high consumption of preserved foods and insufficient intake of fresh fruits and vegetables (23–25). In the present study, we specifically focused on high-sodium diet as a major risk factor. This choice was primarily driven by the availability of detailed and standardized data in the GBD 2021 database, which enabled systematic analyses and reliable trend projections. Moreover, high sodium intake represents a globally prevalent dietary pattern, and its carcinogenic effect on GC has been consistently demonstrated in previous epidemiological and experimental studies. By concentrating on this modifiable risk factor, our study aims to provide actionable evidence and more concrete intervention targets for public health policymaking.

Studies have shown that a high-sodium diet significantly affects the therapeutic outcomes of certain cancers (26–29). Evidence suggests that in GC patients, excessive sodium intake may exacerbate edema and the risk of hypertension, irritate the gastrointestinal tract, and potentially increase the risk of tumor recurrence (8). Furthermore, although this study reveals an association between high sodium intake and GC risk, the underlying biological mechanisms remain to be elucidated. A key future direction will be to actively explore the role of sodium metabolism pathways in the translational application of liquid biopsy technologies. By focusing on the analysis of ctDNA mutational profiles, fragmentomics features, exosomal RNA, and microbe-derived cfRNA in patient plasma, we aim to identify specific molecular biomarkers driven by high sodium exposure (30). Our ultimate vision is to integrate these “sodium footprint” biomarkers into a future multi-cancer joint screening system, thereby establishing an early-warning model for GC that incorporates dietary risk factors and is applicable to multinational populations (31). Finally, a high-sodium diet may lead to elevated sodium ion concentrations within the tumor microenvironment (32). Increasing evidence indicates that this localized high-sodium milieu may activate or upregulate voltage-gated sodium channels (VGSCs) on tumor cell membranes (33–35). VGSCs are frequently aberrantly expressed in various cancers, including GC, and their activity is closely linked to the invasive, migratory, and metastatic capabilities of tumor cells (33, 36). Therefore, we hypothesize that dietary sodium intake may, at least in part, modulate sodium levels in the tumor microenvironment, thereby influencing the functional state of VGSCs and ultimately “empowering” tumor cells with enhanced invasive and metastatic potential.

From 1990 to 2021, the global number of DALYs due to GC-DHIS slightly declined, while the number of deaths increased. This seemingly paradoxical phenomenon can be explained by population growth and aging. Although population growth and aging contributed to an absolute increase in GC burden, effective public health interventions and advances in medical technology have led to a decline in ASDR and ASMR. Middle SDI regions had the highest number of DALYs, while the fastest growth occurred in low-middle SDI regions, possibly due to limited health awareness and medical resources during economic development. In low SDI regions, high sodium intake, compounded by scarce medical resources and low health literacy, may further exacerbate the GC burden. In contrast, in high SDI regions, although health awareness and medical resources are more abundant, high sodium dietary habits persist, contributing to relatively high GC incidence. Oceania experienced a sharp increase in DALYs and deaths, while East Asia had the highest ASDR and ASMR, which may be linked to the traditionally high-sodium diet in this region. Overall, despite the global decline in age-standardized rates of GC-DHIS, the burden continues to rise in certain regions and populations. Targeted public health policies are therefore needed, particularly in high-risk regions and populations, to reduce sodium consumption, improve health awareness, and optimize medical resource allocation. These measures are essential to mitigate the GC burden in the context of rapid population growth and aging.

Over the past 32 years, approximately 65% of countries experienced an upward trend in DALYs due to GC-DHIS, and about 70% saw an increase in deaths. Countries such as Djibouti, the United Arab Emirates, and Honduras showed particularly notable increases in DALYs and deaths. Additionally, countries like Mongolia and Bolivia had relatively high ASDR and ASMR, reflecting significant geographic disparities in the impact of high sodium intake on GC. In terms of age and sex, in 2021, the 65–69 age group had the highest number of DALYs, while the 70–74 age group had the highest number of deaths. The highest ASDR was recorded in females aged 95+ and males aged 85–89, whereas the highest ASMR was found in females aged 95+ and males aged 90–94. These findings suggest that older populations are more severely affected by the consequences of high sodium intake, and the burden of GC is particularly pronounced among elderly males.

Decomposition analysis revealed that while the global number of DALYs due to GC-DHIS has decreased overall, DALYs among males have increased, whereas those among females have declined significantly. Meanwhile, the number of death cases has shown an upward trend, with the increase being more pronounced in males than in females. These changes have been significantly influenced by population aging, growth, and epidemiological changes. Aging and population growth have contributed to the increase in DALYs and death cases, while epidemiological changes have partially mitigated this upward trend.

Health inequality analysis indicated that although the absolute disparity between countries with the highest and lowest SDI levels has narrowed, the disease burden remains more concentrated in higher SDI regions. This may be attributed to factors such as high-sodium dietary habits, and lifestyle patterns in these more developed areas.

Forecasting analysis indicated that by 2045, the absolute burden of GC-DHIS will continue to rise, with men experiencing more DALYs and deaths than women. This disparity may stem from differences in exposure levels, biological factors, and access to healthcare (37, 38). First, men may consume more sodium than women due to dietary preferences, such as a greater tendency to consume salty foods. Second, biological differences, including hormone levels and gene expression, may influence GC development differently in men and women. Finally, men may be less likely than women to participate in regular health checkups and cancer screening, leading to delayed diagnosis and treatment, which in turn contributes to higher disease severity and mortality. Meanwhile, ASDR and ASMR are projected to continue declining, suggesting that despite population pressures, the population-level risk is decreasing. These findings underscore the urgency of implementing targeted interventions for high-risk male populations, while continuing broader efforts to reduce dietary sodium intake. However, it should be noted that the forecasting model assumes no major policy or intervention changes in the coming decades. If effective public health interventions are implemented in the future—such as sodium reduction policies and health awareness campaigns—the actual burden may be lower than predicted.

This study also has several limitations. First, there may be inconsistencies in the definition and quantification of high-sodium diets. Dietary habits vary across countries and regions, leading to potential discrepancies in how “high-sodium diet” is defined, which could introduce bias in cross-regional comparisons. Second, although the study covers 204 countries and territories, data from some small countries or regions may be incomplete or not fully representative, potentially affecting the global representativeness of the findings.

## Conclusion

This study, based on the GBD 2021 database, analyzed the global and regional burden of GC-DHIS from 1990 to 2021, and projected trends from 2022 to 2045. The results showed that from 1990 to 2021, the global number of DALYs due to GC-DHIS slightly declined, while deaths increased. Middle SDI regions bore the highest disease burden, but the fastest increase occurred in Low-middle SDI regions. Around 65% of countries experienced an increase in DALYs, and approximately 70% saw an increase in deaths, with significant rises observed in Djibouti, the United Arab Emirates, and Honduras. In 2021, the highest number of DALYs occurred in the 65–69 age group, and the highest number of deaths in the 70–74 age group. Additionally, while the absolute inequality gap between countries with the highest and lowest SDI levels has narrowed, the disease burden has become more concentrated in higher-SDI regions. Projections from 2022 to 2045 indicate a significant increase in DALYs and deaths due to GC-DHIS, with faster growth among males, although ASDR and ASMR are expected to decline notably. Therefore, GC-DHIS remains a major global public health concern. Despite the overall decline in ASDR and ASMR, the burden continues to rise in certain regions and populations. More effective health interventions targeting older adults and male populations are needed to address the potentially increasing burden of GC.

## Data Availability

The original contributions presented in the study are included in the article/Supplementary material, further inquiries can be directed to the corresponding author.
