# Supplementary material for: Global, regional, and national burden of gastric cancer attributable to diet high in sodium from 1990 to 2021
Source: Front Nutr. 2025 Oct 7;12:1674979. doi: 10.3389/fnut.2025.1674979 (PMC12537357; doi:10.3389/fnut.2025.1674979)
Supplement: Supplementary file 1 [file Data_Sheet_1.PDF]

Location	1990_DALYs cases (95% UI)	2021_DALYs cases (95% UI)	Percentage change	1990_ASDR_per 100000(95% UI)	2021_ASDR_per 100 000(95% UI)	EAPC (95% CI)
Afghanistan	4107.66 (0-25272.02)	5393.22 (0-34027.76)	0.31	57.31 (0-356.12)	42.9 (0-275.01)	-1.16 (-1.41--0.91)
Albania	716.14 (0-3578.23)	808.94 (0-4220.13)	0.13	33.44 (0-168.87)	19.05 (0-99.74)	-1.7 (-1.94--1.47)
Algeria	1004.27 (0-5941.82)	1595.65 (0-9529.45)	0.59	7.63 (0-45.49)	4.22 (0-25.39)	-1.87 (-1.97--1.78)
American Samoa	8.37 (0-48.22)	13.63 (0-75.33)	0.63	33.5 (0-190.16)	28.69 (0-156.55)	-0.43 (-0.52--0.35)
Andorra	11.51 (0-62.58)	14.79 (0-85.56)	0.28	19.76 (0-108.15)	9.75 (0-56.37)	-2.03 (-2.26--1.79)
Angola	996.35 (0-6061.17)	1937.66 (0-11242.83)	0.94	22.24 (0-135.36)	14.38 (0-81.79)	-1.6 (-1.67--1.53)
Antigua and Barbuda	15.93 (0-81.4)	17.44 (0-92.86)	0.09	30.31 (0-154.49)	16.16 (0-85.68)	-2 (-2.16--1.84)
Argentina	8635.26 (0-44565.68)	8671.63 (0-43935.95)	0	26.69 (0-137.72)	15.85 (0-80.22)	-1.42 (-1.58--1.27)
Armenia	1385.93 (0-6883.17)	855.1 (0-4465.05)	-0.38	47.39 (0-234.69)	20.07 (0-104.48)	-2.53 (-2.73--2.33)
Australia	1975.79 (0-10968.86)	2197.82 (0-12383.02)	0.11	10.26 (0-56.75)	5.32 (0-29.71)	-2.07 (-2.15--1.99)
Austria	3199.23 (0-16155.7)	1368.33 (0-6945.1)	-0.57	27.72 (0-140.14)	8.04 (0-40.82)	-3.86 (-4.04--3.68)
Azerbaijan	3020.72 (0-14868.37)	2983.24 (0-15762.48)	-0.01	55.91 (0-276.11)	27.11 (0-143.45)	-2.45 (-2.59--2.31)
Bahrain	32.45 (0-191.7)	69.21 (0-409.77)	1.13	16.49 (0-96.66)	6.92 (0-42.21)	-3.38 (-3.69--3.07)
Bangladesh	10608.08	14956.14	0.41	20.37	10.42	-2.01

	(0-54919.03)	(0-77504.74)		(0-104.95)	(0-53.93)	(-2.18--1.83)
Barbados	75.12 (0-401.99)	72.2 (0-409.66)	-0.04	26.59 (0-142.59)	14.46 (0-82.29)	-1.93 (-2.1--1.76)
Belarus	7645.34 (0-41414.85)	3429.09 (0-18075.48)	-0.55	59.39 (0-320.65)	22.55 (0-118.46)	-3.76 (-3.97--3.54)
Belgium	2947.07 (0-14810.64)	1649.28 (0-8401.05)	-0.44	19.59 (0-98.23)	7.56 (0-38.48)	-2.95 (-3.1--2.81)
Belize	20.56 (0-107.74)	52.73 (0-276.1)	1.56	21.56 (0-112.75)	16.51 (0-86.39)	-1.04 (-1.59--0.48)
Benin	467.97 (0-2485.41)	918.36 (0-4782)	0.96	22.57 (0-119.19)	16.67 (0-86.71)	-0.75 (-0.92--0.58)
Bermuda	14.12 (0-73.77)	10.33 (0-55.77)	-0.27	22.38 (0-116.93)	7.89 (0-42.51)	-3.23 (-3.46--2.99)
Bhutan	47.56 (0-254.34)	66.42 (0-362.42)	0.4	16.61 (0-88.33)	10.49 (0-57.2)	-1.41 (-1.54--1.29)
Bolivia	3285.83 (0-16547.44)	5259.45 (0-26842.82)	0.6	97.64 (0-491.71)	56.47 (0-288.2)	-1.85 (-1.93--1.77)
Bosnia and Herzegovina	1219.72 (0-5918.64)	1006.6 (0-5168.79)	-0.17	28.17 (0-137.3)	16.63 (0-84.94)	-1.89 (-2.06--1.73)
Botswana	112.8 (0-641.02)	142.4 (0-796.1)	0.26	18.35 (0-104.67)	8.62 (0-48.6)	-2.58 (-2.82--2.33)
Brazil	35485.29 (0-177282.94)	48259.56 (0-247638.54)	0.36	37.77 (0-188.6)	18.95 (0-97.31)	-2.35 (-2.41--2.3)
Brunei	57.56 (0-290.48)	76.66 (0-387.69)	0.33	46.76 (0-237.2)	19.53 (0-98.37)	-2.73 (-3.01--2.44)
Bulgaria	5507.61 (0-27298.42)	2987.63 (0-14705.48)	-0.46	44.89 (0-222.56)	22.76 (0-112.13)	-1.94 (-2.11--1.76)
Burkina Faso	1120 (0-6133.64)	1968.31 (0-11098.15)	0.76	24.67 (0-134.44)	20.06 (0-112.79)	-0.36 (-0.55--0.17)

Burundi	681.99 (0-3576.92)	822.81 (0-4343.09)	0.21	26.97 (0-140.64)	14.94 (0-77.39)	-2.46 (-2.68- -2.24)
Cambodia	1866.89 (0-10015.85)	2814.24 (0-13956.35)	0.51	36.7 (0-195.64)	21.06 (0-104.42)	-1.98 (-2.08- -1.88)
Cameroon	1053.61 (0-5691.63)	2438.45 (0-13966.93)	1.31	22.07 (0-118.96)	17.73 (0-100.65)	-0.51 (-0.64- -0.37)
Canada	4334.29 (0-22114.61)	4428.28 (0-22544.62)	0.02	13.45 (0-68.65)	6.63 (0-33.55)	-2.13 (-2.23- -2.03)
Cape Verde	117.76 (0-622.1)	169.91 (0-922.31)	0.44	52.51 (0-276.31)	37.72 (0-205.61)	-1.29 (-1.72- -0.85)
Central African Republic	375.4 (0-2129.13)	609.72 (0-3521.55)	0.62	28.67 (0-162.1)	22.89 (0-132.24)	-0.84 (-0.92- -0.75)
Chad	605.68 (0-3271.74)	1472.66 (0-8136.27)	1.43	20.84 (0-112.96)	23.57 (0-130.09)	0.61 (0.48-0 .74)
Chile	6471.89 (0-32468.77)	6894.09 (0-34596.97)	0.07	63.24 (0-317.68)	27.13 (0-135.98)	-2.63 (-2.72- -2.55)
China	892814.81 (-0.04-4340 139.74)	883434.61 (-0.01-4461 210.86)	-0.01	98.4 (0-478.5)	41.46 (0-208.59)	-2.91 (-3.1-- 2.72)
Colombia	11274.12 (0-56725.45)	14939.75 (0-76562.64)	0.33	60.69 (0-303.31)	27.16 (0-139.11)	-2.92 (-3.07- -2.77)
Comoros	44.63 (0-236.59)	68.26 (0-357.32)	0.53	20.45 (0-108.08)	13.11 (0-68.4)	-1.72 (-1.87- -1.56)
Congo	277.62 (0-1607.26)	452.14 (0-2618.31)	0.63	23.83 (0-138.15)	14.53 (0-82.32)	-1.9 (-2.05- -1.74)
Cook Islands	2.63 (0-13.82)	2.76 (0-14.08)	0.05	20.67 (0-107.53)	11.12 (0-57.42)	-1.91 (-2.08- -1.74)
Costa Rica	1218.41 (0-6028.4)	1765.74 (0-8912.06)	0.45	67.48 (0-334.34)	32.07 (0-161.83)	-2.78 (-3--2. 55)
Cote d'Ivoire	374.28 (0-1936.17)	787.35 (0-4235.35)	1.1	8.23 (0-42.67)	6.17 (0-32.87)	-0.88 (-1.06-

						-0.69)
Croatia	2762.32 (0-14012.87)	1323.94 (0-6552.54)	-0.52	45.24 (0-228.51)	15.64 (0-77.58)	-3.4 (-3.51--3.29)
Cuba	1385.56 (0-7285.69)	1805.16 (0-9312.97)	0.3	13.48 (0-70.86)	9.47 (0-48.88)	-1.01 (-1.11--0.9)
Cyprus	126.65 (0-665.33)	168.89 (0-889.43)	0.33	16.99 (0-88.57)	8.45 (0-44.6)	-1.88 (-2.02--1.75)
Czech Republic	4404.74 (0-21833.74)	2087.77 (0-10355.22)	-0.53	32.21 (0-159.49)	10.23 (0-50.64)	-3.79 (-3.86--3.72)
Democratic People's Republic of Korea	10912.67 (0-54774.63)	16585.37 (0-85026.12)	0.52	61.06 (0-305.53)	48.47 (0-249.15)	-0.77 (-0.88--0.66)
Democratic Republic of the Congo	2422.36 (0-14459.63)	4659.05 (0-28099.42)	0.92	14.1 (0-83.72)	11.2 (0-67.87)	-0.7 (-0.75--0.65)
Denmark	1209.25 (0-6296.34)	851.45 (0-4472.53)	-0.3	15.96 (0-82.73)	7.71 (0-40.23)	-2.21 (-2.48--1.95)
Djibouti	30.3 (0-160.91)	101.19 (0-557.17)	2.34	18.73 (0-97.98)	13.99 (0-74.78)	-1.14 (-1.29--0.98)
Dominica	28.26 (0-148.58)	27.86 (0-146.64)	-0.01	48.02 (0-252.12)	33.29 (0-175.28)	-1.22 (-1.36--1.08)
Dominican Republic	584.09 (0-3101.89)	1277.72 (0-6775.91)	1.19	14.82 (0-78.25)	12.45 (0-66.1)	-0.17 (-0.29--0.04)
Ecuador	3201.51 (0-15958.71)	5575.62 (0-28478.25)	0.74	57.91 (0-289.9)	33.62 (0-171.72)	-1.88 (-2.26--1.5)
Egypt	2127.25 (0-12029.59)	6315.05 (0-37758.63)	1.97	6.57 (0-37.37)	8.87 (0-53.2)	2.07 (1.52-2.63)
El Salvador	1237.91 (0-6244.91)	2135.4 (0-10918.73)	0.73	39.75 (0-200.31)	34.85 (0-178.14)	-0.79 (-1.02--0.57)
Equatorial Guinea	52.47 (0-313.94)	61.2 (0-348.31)	0.17	24.64 (0-147.24)	10.35 (0-59.07)	-3.17 (-3.52--2.82)

Eritrea	440.53 (0-2221.78)	641.99 (0-3511.76)	0.46	30.92 (0-160.17)	19.77 (0-107.3)	-1.52 (-1.57- -1.47)
Estonia	778.8 (0-4422.35)	325.06 (0-1819.02)	-0.58	38.48 (0-218.65)	13.42 (0-74.98)	-3.66 (-3.79- -3.53)
Ethiopia	8247.58 (0-41968.48)	6183.29 (0-33133.62)	-0.25	35.48 (0-180.82)	12.63 (0-67.11)	-3.9 (-4.12- -3.68)
Fiji	75.73 (0-436.09)	115.21 (0-627.65)	0.52	19.08 (0-106.74)	14.67 (0-79.24)	-0.83 (-1.02- -0.64)
Finland	1525.62 (0-8006.2)	766.17 (0-3995.95)	-0.5	21.97 (0-114.77)	6.68 (0-34.87)	-3.69 (-3.81- -3.57)
France	12978.76 (0-68947.18)	9345.2 (0-49117.16)	-0.28	16.14 (0-85.72)	7.47 (0-39.32)	-2.19 (-2.29- -2.1)
Gabon	112.41 (0-645.09)	143.73 (0-824.65)	0.28	19.19 (0-109.79)	12.75 (0-72.33)	-1.44 (-1.57- -1.31)
Georgia	2406.46 (0-11945.94)	1282.26 (0-6630.55)	-0.47	38.06 (0-188.76)	22.66 (0-117.21)	-0.69 (-1.11- -0.27)
Germany	29254.27 (0-153643.0 4)	17257.39 (0-86397.28)	-0.41	23.9 (0-125.09)	10.09 (0-50.47)	-2.83 (-2.98- -2.68)
Ghana	1187.93 (0-6358.78)	2465.08 (0-12776.36)	1.08	17.48 (0-92.91)	13.81 (0-71.2)	-0.55 (-0.61- -0.49)
Greece	3857.38 (0-20094.04)	2623.83 (0-13614.25)	-0.32	26.03 (0-135.49)	12.22 (0-62.73)	-2.67 (-2.88- -2.46)
Greenland	16.2 (0-82.61)	12.42 (0-64.06)	-0.23	40 (0-203.64)	17 (0-87.85)	-2.75 (-2.87- -2.63)
Grenada	18.1 (0-93.95)	16.54 (0-88.07)	-0.09	26.44 (0-137.7)	14.34 (0-75.83)	-1.74 (-1.96- -1.52)
Guam	12.29 (0-64.9)	21.56 (0-113.23)	0.75	15.67 (0-80.55)	10.64 (0-56.35)	-0.85 (-1.23- -0.47)
Guatemala	2174.09 (0-10800.57	5396.09 (0-27892.64	1.48	58.91 (0-293.38)	46.77 (0-242.03)	-1.15 (-1.77-

	)	)				-0.53)
Guinea	682.28 (0-3624.44)	1073.61 (0-5838)	0.57	19.73 (0-105.27)	17.44 (0-94.45)	-0.18 (-0.33--0.03)
Guinea-Bissau	159.96 (0-897.51)	239.18 (0-1304.1)	0.5	36.56 (0-203.01)	28.79 (0-155.74)	-0.39 (-0.55--0.22)
Guyana	102.22 (0-531.19)	100.24 (0-561.47)	-0.02	25.37 (0-132.5)	14.74 (0-82.09)	-1.5 (-1.76--1.23)
Haiti	1833.42 (0-9806.13)	2589.59 (0-13905.75)	0.41	52.78 (0-283.13)	32.74 (0-174.87)	-1.4 (-1.5--1.3)
Honduras	797.13 (0-3964.24)	2436.27 (0-12395.82)	2.06	35.34 (0-176.07)	37.28 (0-189.21)	0.32 (0.17-0.47)
Hungary	5708.06 (0-28299.93)	2537.83 (0-12449.69)	-0.56	39.47 (0-196.42)	14.02 (0-68.82)	-3.32 (-3.51--3.13)
Iceland	64.08 (0-327.71)	38.44 (0-201.83)	-0.4	22.95 (0-117.69)	6.92 (0-36.08)	-3.76 (-3.84--3.68)
India	93863.7 (0-482094.85)	149554.57 (0-754254.38)	0.59	17.43 (0-90.46)	11.76 (0-59.4)	-1.16 (-1.27--1.05)
Indonesia	23391.42 (0-122084.03)	37686.19 (0-189056.63)	0.61	20.82 (0-107.86)	14.58 (0-73.62)	-1.11 (-1.18--1.04)
Iran	8781.94 (0-50204.6)	12704.05 (0-74972.66)	0.45	30.43 (0-175.31)	15.55 (0-92.33)	-1.95 (-2.1--1.8)
Iraq	878.52 (0-5160.19)	1799.08 (0-10740.59)	1.05	9.89 (0-58.08)	6.62 (0-39.91)	-1.56 (-1.64--1.47)
Ireland	797.93 (0-4275.28)	503.25 (0-2717.3)	-0.37	19.82 (0-105.93)	6.53 (0-35.12)	-3.56 (-3.66--3.46)
Israel	825.49 (0-4310.31)	978.28 (0-5020.15)	0.19	17.35 (0-90.49)	8.23 (0-42.16)	-2.75 (-2.91--2.59)
Italy	28832.9 (0-144128.48)	16109.46 (0-82405)	-0.44	33.51 (0-166.96)	11.95 (0-60.43)	-3.27 (-3.33--3.21)
Jamaica	460.9	485.77	0.05	26.02	15.73	-1.74

	(0-2376.2)	(0-2648.48)		(0-134.3)	(0-85.8)	(-2.08--1.4)
Japan	110925.63 (0-544308.9)	76536.72 (0-383663.82)	-0.31	65.73 (0-322.56)	22.26 (0-111.56)	-3.52 (-3.57--3.47)
Jordan	155.44 (0-876.77)	396.92 (0-2290.98)	1.55	9.9 (0-55.99)	4.69 (0-27.71)	-2.62 (-2.83--2.4)
Kazakhstan	9937.78 (0-49315.96)	4153.4 (0-21560.52)	-0.58	73.78 (0-365.38)	21.61 (0-112.23)	-3.82 (-4.01--3.64)
Kenya	1143.71 (0-6299.61)	2828.46 (0-16302.13)	1.47	12.73 (0-69.29)	11.21 (0-64.41)	-0.05 (-0.28-0.18)
Kiribati	19.51 (0-107.33)	34.72 (0-188.66)	0.78	47.5 (0-253.3)	42.64 (0-225.77)	-0.35 (-0.41--0.28)
Kuwait	52.36 (0-276.02)	134.37 (0-709.36)	1.57	6.99 (0-37.37)	3.98 (0-21.48)	-2.04 (-2.47--1.6)
Kyrgyzstan	2277.57 (0-11235.3)	1707.16 (0-8951.02)	-0.25	72.74 (0-359.43)	31.59 (0-165.39)	-2.48 (-2.66--2.31)
Lao People's Democratic Republic	902.79 (0-4721.41)	891.88 (0-4530.17)	-0.01	38.99 (0-203.26)	17.38 (0-88.01)	-2.81 (-2.9--2.73)
Latvia	1624.46 (0-8803.88)	682.35 (0-3521.05)	-0.58	46.32 (0-250.83)	19.65 (0-100.56)	-2.98 (-3.14--2.82)
Lebanon	314.01 (0-1844.66)	370.79 (0-2187.64)	0.18	13.7 (0-81.02)	6.22 (0-36.59)	-2.25 (-2.41--2.1)
Lesotho	140.35 (0-775.77)	261.6 (0-1461.84)	0.86	15.89 (0-88.16)	21.79 (0-122.95)	1.76 (1.29-2.24)
Liberia	233.88 (0-1279.56)	407.53 (0-2248.71)	0.74	19.57 (0-106.49)	17.25 (0-95.14)	-0.33 (-0.5--0.16)
Libya	218.78 (0-1322.66)	510.15 (0-3156.18)	1.33	10.54 (0-63.86)	8.46 (0-52.16)	-0.45 (-0.6--0.29)
Lithuania	2129.13 (0-11206.69)	1027.23 (0-5280.8)	-0.52	47.84 (0-251.53)	20.24 (0-104.25)	-2.73 (-2.87--2.6)

Luxembourg	117.04 (0-605.62)	70.28 (0-365.74)	-0.4	22.11 (0-114.17)	6.68 (0-34.69)	-3.74 (-3.82--3.67)
Macedonia	841.25 (0-3988.13)	825.04 (0-4042.65)	-0.02	43.73 (0-207.42)	24.91 (0-122.52)	-2.11 (-2.33--1.89)
Madagascar	1054.66 (0-5693.67)	1651.98 (0-8910.05)	0.57	18.8 (0-100.96)	12.5 (0-64.66)	-1.4 (-1.46--1.33)
Malawi	356.72 (0-1774.66)	515.11 (0-2670.45)	0.44	8.34 (0-41.56)	6.21 (0-31.89)	-1.32 (-1.6--1.04)
Malaysia	1809.53 (0-8919.42)	3613.41 (0-18386.5)	1	18.2 (0-90.4)	12.29 (0-62.33)	-1.41 (-1.56--1.27)
Maldives	20.99 (0-107.74)	19.69 (0-101.28)	-0.06	20.52 (0-105.03)	5.06 (0-25.89)	-4.94 (-5.2--4.68)
Mali	1790.67 (0-9508.04)	2972.59 (0-15640.41)	0.66	41.04 (0-219.42)	30.42 (0-159.74)	-0.73 (-0.85--0.62)
Malta	96.33 (0-490.04)	71.89 (0-365.54)	-0.25	22.49 (0-114.32)	7.88 (0-40.12)	-3.2 (-3.33--3.07)
Marshall Islands	8.11 (0-43.97)	14.04 (0-75.43)	0.73	44.72 (0-232.18)	34.9 (0-184.28)	-0.74 (-0.83--0.64)
Mauritania	222.7 (0-1199.22)	325.67 (0-1762.36)	0.46	21.58 (0-115.65)	14.69 (0-79.36)	-1.1 (-1.36--0.85)
Mauritius	257.17 (0-1299.03)	375.84 (0-1858.13)	0.46	32.77 (0-165.01)	20.67 (0-102.35)	-2.25 (-2.64--1.87)
Mexico	12327.83 (0-63326.48)	22345.98 (0-116943.2)	0.81	27.82 (0-142.03)	17.14 (0-89.36)	-1.74 (-1.87--1.6)
Micronesia (Federated States of)	23.1 (0-125.95)	26.83 (0-142.61)	0.16	44.15 (0-237.28)	33.3 (0-175.38)	-0.93 (-1.03--0.84)
Moldova	1771.1 (0-9554.79)	908.99 (0-4912.81)	-0.49	38.48 (0-207.59)	15.63 (0-84.42)	-2.38 (-2.72--2.05)
Monaco	12.51 (0-65.14)	10.04 (0-53.39)	-0.2	19.69 (0-102.73)	11.58 (0-60.31)	-1.71 (-1.83-

						-1.59)
Mongolia	1297.34 (0-6469.78)	1903.76 (0-9842.87)	0.47	116.56 (0-576.28)	73.19 (0-380.58)	-1.89 (-2.06--1.73)
Montenegro	111.86 (0-542.21)	135.33 (0-680.23)	0.21	17.54 (0-84.88)	14.08 (0-70.68)	-0.84 (-1.08--0.61)
Morocco	675.3 (0-3875.27)	1135.29 (0-6797.13)	0.68	4.4 (0-25.34)	3.14 (0-18.98)	-1.02 (-1.08--0.96)
Mozambique	954.08 (0-4645.9)	1632.83 (0-8773.08)	0.71	15.65 (0-75.82)	14.21 (0-74.86)	0.14 (-0.04-0.32)
Myanmar	9092.18 (0-47681.33)	7695.1 (0-39007.43)	-0.15	35.23 (0-183.22)	14.98 (0-75.45)	-3.16 (-3.3--3.01)
Namibia	44.82 (0-240.88)	72.49 (0-395.93)	0.62	6.22 (0-33.55)	4.58 (0-25)	-1.21 (-1.56--0.86)
Nauru	2.7 (0-14.51)	2.78 (0-15.22)	0.03	51.4 (0-269.18)	41.59 (0-217.06)	-0.79 (-1.07--0.51)
Nepal	1882.57 (0-10103.94)	2951.23 (0-15051.1)	0.57	17.54 (0-93.06)	12.08 (0-61.32)	-1.01 (-1.3--0.73)
Netherlands	3775.92 (0-20124.92)	2355.78 (0-12387.86)	-0.38	19.33 (0-102.6)	7.05 (0-36.75)	-3.22 (-3.35--3.1)
New Zealand	627.1 (0-3277.56)	611.07 (0-3275.06)	-0.03	16.26 (0-84.84)	7.79 (0-41.76)	-2.45 (-2.69--2.2)
Nicaragua	458.41 (0-2334.71)	979.37 (0-4999.73)	1.14	27.27 (0-138.44)	18.95 (0-96.48)	-1.13 (-1.36--0.89)
Niger	717.96 (0-3877.54)	1673.82 (0-9215.06)	1.33	23.55 (0-126.82)	19.28 (0-105.25)	-0.33 (-0.51--0.16)
Nigeria	2934.12 (0-15756.73)	4072.46 (0-22551.03)	0.39	6.24 (0-33.69)	4.11 (0-22.85)	-1.34 (-1.45--1.23)
Niue	0.57 (0-2.87)	0.44 (0-2.25)	-0.23	26.61 (0-133.54)	20.96 (0-106.92)	-1.01 (-1.08--0.94)
Northern	6.45	12.73	0.97	30.52	24.14	-0.66

Mariana Islands	(0-35.48)	(0-64.41)		(0-162.34)	(0-123.29)	(-0.94--0.38)
Norway	1103.06 (0-5818.31)	524.64 (0-2791.2)	-0.52	17.2 (0-90.45)	5.45 (0-28.98)	-3.7 (-3.76--3.64)
Oman	125.26 (0-714.91)	155.27 (0-884.33)	0.24	15.64 (0-91.83)	6.41 (0-37.75)	-2.55 (-2.69--2.41)
Pakistan	6169.28 (0-31945.2)	13277.71 (0-65439.03)	1.15	10.26 (0-53.22)	9.51 (0-46.81)	-0.47 (-0.76--0.18)
Palau	4.73 (0-25.72)	7.95 (0-41.98)	0.68	45.69 (0-245.01)	35 (0-183.43)	-0.74 (-0.8--0.68)
Palestine	131.6 (0-805.71)	204.58 (0-1203.88)	0.55	14.49 (0-89.29)	7.37 (0-43.42)	-2.26 (-2.47--2.05)
Panama	410.44 (0-2045.23)	833.27 (0-4404.98)	1.03	26.59 (0-132.63)	18.87 (0-99.69)	-1.13 (-1.28--0.98)
Papua New Guinea	694.64 (0-3899.29)	1530.13 (0-8512.14)	1.2	34.69 (0-189.6)	26.74 (0-145.56)	-0.87 (-0.92--0.82)
Paraguay	454.3 (0-2320.64)	963.44 (0-5024.82)	1.12	19.72 (0-100.22)	16.13 (0-83.9)	-0.94 (-1.17--0.72)
Peru	6922.63 (0-35443.33)	12292.62 (0-63937.65)	0.78	54.88 (0-280.51)	36.04 (0-187.32)	-1.7 (-1.97--1.44)
Philippines	4082.19 (0-20610.02)	8288.36 (0-42711.68)	1.03	11.82 (0-59.59)	9.21 (0-47.21)	-0.72 (-0.81--0.64)
Poland	17532.05 (0-87991.98)	10989.52 (0-54420.59)	-0.37	40.11 (0-201.26)	16.25 (0-80.58)	-2.99 (-3.13--2.86)
Portugal	6252.58 (0-33605.87)	3959.85 (0-20796.83)	-0.37	47.01 (0-252.12)	18.46 (0-94.95)	-2.93 (-3.03--2.83)
Puerto Rico	721.42 (0-3732.7)	470.19 (0-2477.25)	-0.35	19.94 (0-103.16)	7.39 (0-39.46)	-3.46 (-3.6--3.32)
Qatar	25.41 (0-145.04)	77.5 (0-435.74)	2.05	18.82 (0-107.01)	6.17 (0-36.75)	-3.75 (-4.32--3.19)

Romania	9271.47 (0-47871.98)	7321.75 (0-36508.17)	-0.21	32.75 (0-168.97)	21.42 (0-106.86)	-1.58 (-1.73--1.42)
Russian Federation	123581.04 (0-628901.09)	58559.82 (0-294977.13)	-0.53	67.29 (0-342.55)	25.26 (0-127.12)	-3.39 (-3.51--3.26)
Rwanda	939.26 (0-4804.61)	888.81 (0-4708.2)	-0.05	29.26 (0-148.19)	12.83 (0-66.63)	-3.8 (-4.22--3.39)
Saint Kitts and Nevis	10.89 (0-55.82)	10.51 (0-58.47)	-0.03	30.25 (0-155.63)	14.9 (0-82.49)	-2.05 (-2.25--1.85)
Saint Lucia	32.5 (0-171.45)	43.98 (0-244.31)	0.35	37.52 (0-197.51)	18.38 (0-102.01)	-2.64 (-2.88--2.41)
Saint Vincent and the Grenadines	22.29 (0-115.18)	25.59 (0-136.97)	0.15	31.36 (0-161.79)	18.1 (0-96.88)	-1.77 (-2--1.55)
Samoa	13.95 (0-86.38)	20.68 (0-125.03)	0.48	16.43 (0-99.62)	14.28 (0-85.36)	-0.49 (-0.6--0.38)
San Marino	14.36 (0-74.34)	10.46 (0-58.57)	-0.27	41.88 (0-218.39)	14.9 (0-83.83)	-2.55 (-2.84--2.26)
Sao Tome and Principe	17.63 (0-93)	27.36 (0-139.85)	0.55	27.16 (0-143.37)	24.09 (0-124.39)	-0.44 (-0.63--0.26)
Saudi Arabia	591.28 (0-3526.56)	1260.98 (0-7212.62)	1.13	8.68 (0-52.7)	4.7 (0-27.44)	-2.02 (-2.19--1.85)
Senegal	744.7 (0-4014.97)	1442.8 (0-8003.64)	0.94	21.85 (0-117.75)	17.81 (0-98.07)	-0.37 (-0.6--0.14)
Serbia	3027.22 (0-15051.01)	2370.37 (0-11740.52)	-0.22	27 (0-134.19)	15 (0-74.13)	-2.29 (-2.53--2.05)
Seychelles	12.36 (0-63.18)	13.84 (0-69.63)	0.12	22.14 (0-112.86)	11.35 (0-57.09)	-2.04 (-2.18--1.91)
Sierra Leone	419.36 (0-2318.9)	727.73 (0-4055.91)	0.74	19.86 (0-109.52)	17.95 (0-99.25)	0.15 (-0.05-0.35)
Singapore	759.71 (0-3683.69)	646.05 (0-3206.59)	-0.15	32.17 (0-157.39)	7.62 (0-37.78)	-4.65 (-4.89-

						-4.4)
Slovakia	2178.21 (0-10668.47)	1450.58 (0-7270.11)	-0.33	36.53 (0-178.7)	15.65 (0-78.59)	-2.66 (-2.76--2.56)
Slovenia	936.74 (0-4618.08)	507.54 (0-2525.02)	-0.46	38.05 (0-187.63)	11.93 (0-59.18)	-3.88 (-4--3.76)
Solomon Islands	71.71 (0-415.13)	154.52 (0-847.63)	1.15	47.35 (0-266.68)	38.27 (0-203.86)	-0.63 (-0.75--0.51)
Somalia	930.74 (0-4781.52)	1762.21 (0-9243.55)	0.89	31.52 (0-159.2)	23.87 (0-123.47)	-1.05 (-1.13--0.97)
South Africa	3160.98 (0-17148.6)	4637.66 (0-25751.95)	0.47	13.44 (0-73.49)	9.27 (0-51.66)	-1.4 (-1.68--1.11)
South Korea	40195.08 (0-196098.1)	21757.16 (0-106997.82)	-0.46	118.73 (0-580.64)	24.08 (0-117.95)	-5.43 (-5.55--5.3)
South Sudan	614.99 (0-3225.42)	809.73 (0-4325.36)	0.32	22.53 (0-117.5)	18.49 (0-97)	-0.91 (-1.12--0.7)
Spain	11552.72 (0-65693.17)	7483.95 (0-41726.26)	-0.35	22.46 (0-125.38)	8.78 (0-47.77)	-2.87 (-2.98--2.76)
Sri Lanka	2067.35 (0-10071.1)	1961.79 (0-10182.93)	-0.05	17.66 (0-86.39)	7.18 (0-37.42)	-3.06 (-3.3--2.83)
Sudan	3034.57 (0-17978.01)	4404.26 (0-27599.61)	0.45	29.18 (0-174.63)	19.54 (0-123.65)	-1.36 (-1.41--1.3)
Suriname	53.55 (0-289.04)	81.69 (0-444.7)	0.53	20.27 (0-108.85)	12.64 (0-69.06)	-1.45 (-1.68--1.23)
Swaziland	63.67 (0-355.8)	109.57 (0-620.89)	0.72	19.66 (0-111.25)	16.45 (0-93.58)	-0.12 (-0.75-0.52)
Sweden	2167.68 (0-11258.3)	1054.57 (0-5509.19)	-0.51	15.21 (0-78.16)	5.16 (0-26.96)	-3.49 (-3.65--3.33)
Switzerland	1765.94 (0-9241.54)	1034.35 (0-5449.95)	-0.41	17.73 (0-92.24)	6.11 (0-31.85)	-3.11 (-3.44--2.78)
Syria	646.01	1000.43	0.55	10.94	7.23 (0-42.5)	-1.57

	(0-3784.23)	(0-5849.05)		(0-65.25)		(-1.74--1.4)
Taiwan (Province of China)	6438.51 (0-32661.6)	6400.41 (0-32826.07)	-0.01	38.17 (0-193.09)	15.52 (0-79.6)	-3.3 (-3.49--3.11)
Tajikistan	1823.17 (0-9210.45)	1864.36 (0-9972.53)	0.02	62.3 (0-314.16)	28.12 (0-150.18)	-2.57 (-2.76--2.38)
Tanzania	2294.88 (0-11354.6)	3414.05 (0-17274.35)	0.49	19.44 (0-95.7)	12.12 (0-60.89)	-1.74 (-1.83--1.65)
Thailand	9566.49 (0-48203.12)	16593.31 (0-88976.09)	0.73	24.17 (0-121.39)	16.02 (0-85.4)	-1.73 (-1.9--1.55)
The Bahamas	43.06 (0-230.82)	66.58 (0-365.11)	0.55	25.98 (0-138.09)	15.77 (0-86.67)	-1.59 (-1.72--1.46)
The Gambia	29 (0-160.69)	66.54 (0-355.84)	1.29	7.48 (0-41.06)	6.22 (0-33.07)	-0.66 (-0.82--0.5)
Timor-Leste	76.11 (0-400.94)	135.72 (0-701.86)	0.78	22.35 (0-116.99)	15.32 (0-78.81)	-1.15 (-1.43--0.87)
Togo	289.39 (0-1560.6)	852.55 (0-4937.1)	1.95	21.31 (0-114.32)	20.31 (0-115.37)	0.16 (0-0.32)
Tokelau	0.43 (0-2.36)	0.29 (0-1.5)	-0.33	32.03 (0-175.75)	19.87 (0-102.66)	-1.68 (-1.72--1.64)
Tonga	21.45 (0-115.67)	24.79 (0-130.01)	0.16	37.87 (0-202.85)	30.56 (0-159.75)	-0.63 (-0.73--0.52)
Trinidad and Tobago	167.45 (0-882.82)	180.33 (0-939.78)	0.08	19.65 (0-103.45)	9.44 (0-49.15)	-2.57 (-2.79--2.35)
Tunisia	450.64 (0-2606.25)	726.37 (0-4396.11)	0.61	8.39 (0-49.2)	5.29 (0-31.84)	-1.68 (-1.76--1.59)
Turkey	11558.57 (0-69944.72)	12107.11 (0-73874.67)	0.05	29.87 (0-183.06)	12.53 (0-76.78)	-2.98 (-3.32--2.65)
Turkmenistan	989.68 (0-4877.1)	901.32 (0-4762.87)	-0.09	47.49 (0-234.31)	20.09 (0-105.89)	-2.84 (-3.13--2.55)

Tuvalu	3.07 (0-16.9)	3.04 (0-16.17)	-0.01	43.29 (0-237.51)	28.45 (0-151.15)	-1.27 (-1.36- -1.19)
Uganda	1271.31 (0-6468.78)	1961.48 (0-10169.68)	0.54	18.41 (0-93.72)	11.95 (0-61.49)	-2.13 (-2.46- -1.8)
Ukraine	36202.9 (0-197249)	14307.12 (0-79232.61)	-0.6	51.57 (0-280.38)	19.95 (0-110.44)	-3.62 (-3.83- -3.42)
United Arab Emirates	114.36 (0-666.77)	378.54 (0-2228.67)	2.31	19 (0-113.86)	8.12 (0-48.99)	-1.68 (-2.15- -1.21)
United Kingdom	14942.96 (0-81248.86)	7852.96 (0-42733.41)	-0.47	16.6 (0-90.58)	6.22 (0-33.88)	-3.13 (-3.26- -3)
United States	27283.93 (0-143481.6 6)	28189.61 (0-144065.0 5)	0.03	8.92 (0-46.83)	5.38 (0-27.4)	-1.69 (-1.74- -1.63)
Uruguay	1169.7 (0-5920.09)	1032.09 (0-5233.51)	-0.12	30.8 (0-155.66)	20.11 (0-102.22)	-1.48 (-1.57- -1.39)
Uzbekistan	5651.16 (0-28368.28)	4784.89 (0-24425.33)	-0.15	46.42 (0-232.81)	15.99 (0-81.55)	-3.18 (-3.36- -3)
Vanuatu	27.2 (0-153.08)	61.35 (0-332.96)	1.26	39.13 (0-213.73)	31.59 (0-167.94)	-0.85 (-0.91- -0.78)
Venezuela	3898.01 (0-19405.61)	6464.82 (0-33104.25)	0.66	38.29 (0-191.2)	21.23 (0-108.69)	-2.35 (-2.55- -2.16)
Viet Nam	13433.72 (0-68381.58)	17444.78 (0-87541.13)	0.3	32 (0-162.06)	16.32 (0-81.42)	-2.5 (-2.68- -2.31)
Virgin Islands, U.S.	18.55 (0-97.99)	15.86 (0-82.89)	-0.15	20.88 (0-109.62)	9.82 (0-51.14)	-2.28 (-2.45- -2.12)
Yemen	1978.45 (0-12384.15)	3969.49 (0-26028.11)	1.01	34.98 (0-220.66)	24.51 (0-160.97)	-1.4 (-1.51- -1.29)
Zambia	696.95 (0-3609.45)	1166.51 (0-6405.51)	0.67	21.8 (0-112.43)	14.42 (0-77.58)	-1.85 (-2.13- -1.57)
Zimbabwe	908.77 (0-4835.6)	2115.97 (0-11467.2)	1.33	20.87 (0-110.33)	26.08 (0-140.73)	1.38 (0.81-1

.95)

Supplementary table 1. The DALYs of GC-DHIS cases and rates in 1990 and 2021 across 204 countries, and the trends from 1990 to 2021.

Location	1990_death cases (95% UI)	2021_death cases (95% UI)	Percentage change	1990_ASMR_per 100000(95% UI)	2021_ASMR_per 100 000(95% UI)	EAPC (95% CI)
Afghanistan	142.27 (0-885.12)	161.69 (0-1025.44)	0.14	2.07 (0-12.89)	1.59 (0-10.32)	-1.05 (-1.29--0.8)
Albania	28.26 (0-143.19)	38.04 (0-195.97)	0.35	1.47 (0-7.46)	0.88 (0-4.51)	-1.56 (-1.8--1.31)
Algeria	35.67 (0-212.45)	59.67 (0-360.72)	0.67	0.32 (0-1.92)	0.18 (0-1.09)	-1.67 (-1.8--1.54)
American Samoa	0.28 (0-1.6)	0.53 (0-2.81)	0.89	1.46 (0-7.89)	1.26 (0-6.61)	-0.38 (-0.46--0.3)
Andorra	0.49 (0-2.69)	0.73 (0-4.28)	0.49	0.89 (0-4.96)	0.46 (0-2.66)	-1.9 (-2.15--1.65)
Angola	32.15 (0-195.41)	64.32 (0-367.93)	1	0.88 (0-5.3)	0.6 (0-3.35)	-1.44 (-1.51--1.37)
Antigua and Barbuda	0.74 (0-3.78)	0.77 (0-4.05)	0.04	1.33 (0-6.81)	0.76 (0-4.01)	-1.8 (-1.96--1.63)
Argentina	363.06 (0-1853.52)	382.32 (0-1939.5)	0.05	1.15 (0-5.84)	0.68 (0-3.43)	-1.46 (-1.6--1.31)
Armenia	48.05 (0-237.83)	36.42 (0-190)	-0.24	1.76 (0-8.76)	0.84 (0-4.37)	-2.01 (-2.21--1.81)
Australia	85.74 (0-482.29)	109.13 (0-629.46)	0.27	0.44 (0-2.49)	0.23 (0-1.34)	-2.01 (-2.1--1.92)
Austria	161.45 (0-812.4)	71.99 (0-364.5)	-0.55	1.31 (0-6.6)	0.37 (0-1.88)	-3.99 (-4.15--3.82)
Azerbaijan	103.42 (0-512.44)	108.91 (0-579.53)	0.05	2.07 (0-10.26)	1.12 (0-5.95)	-1.94 (-2.09--1.8)
Bahrain	1.12 (0-6.59)	2.37 (0-14.28)	1.12	0.73 (0-4.32)	0.33 (0-1.99)	-3.11 (-3.46--2.77)
Bangladesh	355.64	569.92	0.6	0.75 (0-3.87)	0.43 (0-2.2)	-1.76

	(0-1826.62)	(0-2941.29)				(-1.99--1.52)
Barbados	3.61 (0-19.35)	3.42 (0-19.32)	-0.05	1.18 (0-6.36)	0.66 (0-3.73)	-1.89 (-2.1--1.68)
Belarus	280.35 (0-1522.98)	133.8 (0-718.43)	-0.52	2.16 (0-11.74)	0.84 (0-4.51)	-3.59 (-3.8--3.38)
Belgium	152.54 (0-772.53)	90.5 (0-459.54)	-0.41	0.96 (0-4.87)	0.36 (0-1.8)	-3.08 (-3.27--2.88)
Belize	0.86 (0-4.45)	2 (0-10.46)	1.33	0.93 (0-4.82)	0.7 (0-3.66)	-1.11 (-1.67--0.54)
Benin	17.97 (0-95.28)	34.48 (0-179.23)	0.92	0.94 (0-4.96)	0.73 (0-3.79)	-0.55 (-0.72--0.38)
Bermuda	0.61 (0-3.19)	0.54 (0-2.89)	-0.11	1.01 (0-5.26)	0.37 (0-2.01)	-3.11 (-3.34--2.87)
Bhutan	1.51 (0-8.06)	2.62 (0-14.09)	0.74	0.62 (0-3.3)	0.44 (0-2.4)	-0.97 (-1.09--0.85)
Bolivia	124.9 (0-631.16)	218.64 (0-1113.11)	0.75	4.17 (0-21.2)	2.58 (0-13.23)	-1.56 (-1.62--1.5)
Bosnia and Herzegovina	45.24 (0-221.88)	45.87 (0-236.12)	0.01	1.15 (0-5.63)	0.73 (0-3.72)	-1.67 (-1.83--1.5)
Botswana	3.84 (0-21.75)	4.95 (0-27.94)	0.29	0.72 (0-4.04)	0.36 (0-2.01)	-2.34 (-2.55--2.13)
Brazil	1337.64 (0-6704.13)	1948.4 (0-10007.2)	0.46	1.6 (0-8.04)	0.78 (0-4.02)	-2.39 (-2.45--2.34)
Brunei	1.96 (0-9.96)	2.65 (0-13.34)	0.35	1.95 (0-9.92)	0.84 (0-4.3)	-2.5 (-2.77--2.24)
Bulgaria	222.16 (0-1103.95)	134.91 (0-662.15)	-0.39	1.88 (0-9.36)	0.94 (0-4.63)	-1.91 (-2.1--1.73)
Burkina Faso	41.44 (0-222.73)	74.23 (0-417.07)	0.79	1.03 (0-5.5)	0.87 (0-4.86)	-0.21 (-0.38--0.03)

Burundi	23.71 (0-123.54)	28.23 (0-146.49)	0.19	1.04 (0-5.38)	0.62 (0-3.18)	-2.16 (-2.36- -1.96)
Cambodia	61.89 (0-327.47)	101.09 (0-501.79)	0.63	1.39 (0-7.28)	0.86 (0-4.27)	-1.69 (-1.79- -1.59)
Cameroon	37.9 (0-204.78)	87.21 (0-494.51)	1.3	0.92 (0-4.93)	0.76 (0-4.29)	-0.39 (-0.51- -0.26)
Canada	196.21 (0-1015.03)	225.9 (0-1168.07)	0.15	0.6 (0-3.11)	0.3 (0-1.56)	-2.05 (-2.17- -1.94)
Cape Verde	5.43 (0-28.61)	7.52 (0-40.54)	0.38	2.31 (0-12.26)	1.78 (0-9.63)	-1.1 (-1.58- -0.61)
Central African Republic	12.01 (0-68.46)	18.91 (0-109.83)	0.57	1.1 (0-6.27)	0.89 (0-5.12)	-0.77 (-0.85- -0.69)
Chad	23.29 (0-126.12)	53.22 (0-293.53)	1.29	0.86 (0-4.63)	1 (0-5.48)	0.72 (0.59-0 .85)
Chile	269.66 (0-1353.44)	319.43 (0-1605.42)	0.18	2.79 (0-13.97)	1.23 (0-6.18)	-2.54 (-2.65- -2.43)
China	31208.21 (0-152475. 63)	36957.67 (0-183972. 3)	0.18	3.85 (0-18.79)	1.78 (0-8.81)	-2.56 (-2.77- -2.35)
Colombia	432.48 (0-2145.72)	615.87 (0-3149.04)	0.42	2.61 (0-12.88)	1.12 (0-5.7)	-3.13 (-3.27- -3)
Comoros	1.52 (0-8.01)	2.55 (0-13.19)	0.68	0.8 (0-4.14)	0.55 (0-2.83)	-1.42 (-1.54- -1.3)
Congo	9.33 (0-54.52)	15.02 (0-85.41)	0.61	0.94 (0-5.41)	0.61 (0-3.39)	-1.68 (-1.82- -1.55)
Cook Islands	0.1 (0-0.53)	0.12 (0-0.62)	0.2	0.93 (0-4.74)	0.49 (0-2.48)	-2 (-2.13- -1.87)
Costa Rica	50.49 (0-250.12)	74.74 (0-378.62)	0.48	2.97 (0-14.7)	1.36 (0-6.87)	-2.83 (-3.04- -2.62)
Cote d'Ivoire	12.42 (0-64.27)	26.81 (0-143.01)	1.16	0.34 (0-1.78)	0.26 (0-1.35)	-0.81 (-0.99-

						-0.63)
Croatia	116.54 (0-588.12)	66.37 (0-329.7)	-0.43	2.01 (0-10.08)	0.71 (0-3.53)	-3.29 (-3.4--3.17)
Cuba	61.04 (0-316.67)	82.88 (0-428.8)	0.36	0.6 (0-3.14)	0.42 (0-2.15)	-1.02 (-1.13--0.91)
Cyprus	5.84 (0-30.03)	8.59 (0-45.37)	0.47	0.89 (0-4.54)	0.44 (0-2.31)	-1.88 (-2.09--1.68)
Czech Republic	198.11 (0-982.08)	101.53 (0-503.51)	-0.49	1.42 (0-7.02)	0.46 (0-2.26)	-3.85 (-3.95--3.75)
Democratic People's Republic of Korea	366.64 (0-1838.87)	594.16 (0-3058.4)	0.62	2.3 (0-11.52)	1.8 (0-9.26)	-0.79 (-0.91--0.67)
Democratic Republic of the Congo	82.44 (0-489.17)	156.52 (0-949.84)	0.9	0.57 (0-3.4)	0.46 (0-2.79)	-0.69 (-0.73--0.64)
Denmark	56.53 (0-294.32)	42.99 (0-225.58)	-0.24	0.69 (0-3.57)	0.35 (0-1.83)	-2.05 (-2.34--1.77)
Djibouti	0.97 (0-5.09)	3.43 (0-18.44)	2.54	0.75 (0-3.87)	0.6 (0-3.11)	-0.93 (-1.06--0.8)
Dominica	1.32 (0-6.89)	1.22 (0-6.44)	-0.08	2.21 (0-11.61)	1.51 (0-7.98)	-1.32 (-1.44--1.2)
Dominican Republic	21.85 (0-115.42)	51.06 (0-273.44)	1.34	0.64 (0-3.38)	0.52 (0-2.76)	-0.28 (-0.44--0.12)
Ecuador	127.97 (0-636.87)	238.24 (0-1216.71)	0.86	2.55 (0-12.69)	1.5 (0-7.64)	-1.81 (-2.22--1.4)
Egypt	66.51 (0-377.23)	216.16 (0-1297.48)	2.25	0.24 (0-1.4)	0.36 (0-2.16)	2.49 (1.88-3.1)
El Salvador	47.21 (0-238.16)	89.86 (0-456.53)	0.9	1.61 (0-8.13)	1.42 (0-7.23)	-0.77 (-1--0.54)
Equatorial Guinea	1.77 (0-10.64)	2.08 (0-11.82)	0.18	0.97 (0-5.72)	0.45 (0-2.52)	-2.81 (-3.12--2.5)

Eritrea	13.11 (0-67.49)	20.78 (0-113.01)	0.59	1.14 (0-5.88)	0.8 (0-4.29)	-1.2 (-1.24- -1.16)
Estonia	29.7 (0-170.26)	15.23 (0-86.73)	-0.49	1.45 (0-8.32)	0.56 (0-3.14)	-3.29 (-3.41- -3.18)
Ethiopia	260.39 (0-1327.44)	218.73 (0-1161.71)	-0.16	1.3 (0-6.63)	0.53 (0-2.77)	-3.44 (-3.64- -3.24)
Fiji	2.56 (0-14.33)	4.27 (0-22.9)	0.67	0.81 (0-4.4)	0.65 (0-3.47)	-0.78 (-0.94- -0.61)
Finland	69.06 (0-364.92)	41.52 (0-218.44)	-0.4	0.96 (0-5.05)	0.31 (0-1.6)	-3.58 (-3.74- -3.41)
France	667.69 (0-3541.71)	502.78 (0-2641.2)	-0.25	0.77 (0-4.1)	0.33 (0-1.73)	-2.53 (-2.64- -2.43)
Gabon	4.26 (0-24.52)	5.2 (0-29.53)	0.22	0.79 (0-4.51)	0.55 (0-3.04)	-1.32 (-1.44- -1.19)
Georgia	87.53 (0-434.34)	55.05 (0-281.37)	-0.37	1.4 (0-6.95)	0.92 (0-4.72)	-0.31 (-0.77- 0.16)
Germany	1400.08 (0-7438.8)	882.4 (0-4466.36)	-0.37	1.07 (0-5.68)	0.44 (0-2.2)	-2.99 (-3.16- -2.83)
Ghana	41.82 (0-222.03)	91.16 (0-470.17)	1.18	0.73 (0-3.87)	0.61 (0-3.13)	-0.38 (-0.45- -0.31)
Greece	182.94 (0-959.76)	143.72 (0-752.21)	-0.21	1.22 (0-6.37)	0.55 (0-2.87)	-2.87 (-3.1-- 2.63)
Greenland	0.52 (0-2.63)	0.45 (0-2.35)	-0.13	1.54 (0-7.86)	0.68 (0-3.53)	-2.6 (-2.74- -2.46)
Grenada	0.82 (0-4.23)	0.68 (0-3.58)	-0.17	1.1 (0-5.69)	0.64 (0-3.35)	-1.56 (-1.88- -1.24)
Guam	0.45 (0-2.31)	0.8 (0-4.1)	0.78	0.74 (0-3.74)	0.38 (0-1.96)	-1.66 (-2.1-- 1.21)
Guatemala	78.06 (0-388.75)	209.77 (0-1086.93	1.69	2.65 (0-13.21)	1.98 (0-10.25)	-1.3 (-1.95-

		)				-0.64)
Guinea	25.4 (0-135.56)	38.66 (0-208.8)	0.52	0.79 (0-4.23)	0.71 (0-3.81)	-0.13 (-0.27-0.01)
Guinea-Bissau	5.44 (0-30.26)	7.92 (0-42.96)	0.46	1.42 (0-7.8)	1.18 (0-6.36)	-0.18 (-0.35--0.01)
Guyana	3.82 (0-19.82)	3.63 (0-20.16)	-0.05	1.06 (0-5.47)	0.59 (0-3.26)	-1.65 (-1.92--1.39)
Haiti	64.6 (0-345.24)	91.18 (0-486.88)	0.41	2.16 (0-11.4)	1.38 (0-7.33)	-1.32 (-1.41--1.24)
Honduras	28.08 (0-139.39)	97.68 (0-497.8)	2.48	1.43 (0-7.09)	1.68 (0-8.55)	0.69 (0.49-0.89)
Hungary	247.64 (0-1222.86)	116.69 (0-574.66)	-0.53	1.69 (0-8.38)	0.59 (0-2.9)	-3.34 (-3.52--3.16)
Iceland	3.04 (0-15.44)	2.03 (0-10.61)	-0.33	1.04 (0-5.28)	0.33 (0-1.74)	-3.53 (-3.62--3.44)
India	2989.33 (0-15540.99)	5368.18 (0-27163.54)	0.8	0.63 (0-3.29)	0.45 (0-2.31)	-0.91 (-1.04--0.77)
Indonesia	765.13 (0-3955.21)	1352.86 (0-6847.82)	0.77	0.8 (0-4.08)	0.61 (0-3.11)	-0.78 (-0.85--0.71)
Iran	306.53 (0-1754.5)	492.48 (0-2963.78)	0.61	1.23 (0-7.2)	0.66 (0-4.04)	-1.77 (-1.91--1.63)
Iraq	28.83 (0-167.74)	61.63 (0-374.53)	1.14	0.35 (0-2.05)	0.26 (0-1.59)	-1.25 (-1.37--1.13)
Ireland	37.4 (0-200.08)	25.42 (0-138.61)	-0.32	0.91 (0-4.87)	0.31 (0-1.69)	-3.37 (-3.45--3.29)
Israel	38.53 (0-202.27)	49.37 (0-256.16)	0.28	0.82 (0-4.27)	0.39 (0-1.99)	-2.81 (-2.96--2.65)
Italy	1368.94 (0-6948.31)	906.31 (0-4751.75)	-0.34	1.53 (0-7.75)	0.56 (0-2.9)	-3.2 (-3.27--3.13)
Jamaica	21.72	21.63	0	1.19 (0-6.1)	0.69 (0-3.74)	-1.84

	(0-111.72)	(0-117.11)				(-2.15--1.53)
Japan	4680.05 (0-23010.52)	4801.23 (0-24191.03)	0.03	2.81 (0-13.86)	1.09 (0-5.46)	-3.08 (-3.13--3.04)
Jordan	5.02 (0-28.51)	13.82 (0-81.94)	1.75	0.38 (0-2.2)	0.19 (0-1.17)	-2.31 (-2.5--2.11)
Kazakhstan	345.92 (0-1714.22)	149.18 (0-777.57)	-0.57	2.73 (0-13.55)	0.84 (0-4.36)	-3.65 (-3.85--3.45)
Kenya	40.86 (0-221.26)	100.85 (0-580.72)	1.47	0.52 (0-2.79)	0.48 (0-2.73)	0.13 (-0.08-0.34)
Kiribati	0.65 (0-3.42)	1.13 (0-5.92)	0.74	1.89 (0-9.73)	1.73 (0-8.99)	-0.29 (-0.34--0.23)
Kuwait	1.68 (0-8.95)	4.83 (0-26.05)	1.88	0.29 (0-1.56)	0.18 (0-1)	-1.66 (-2.1--1.23)
Kyrgyzstan	76.33 (0-375.31)	57.73 (0-301.68)	-0.24	2.55 (0-12.58)	1.19 (0-6.19)	-2.19 (-2.4--1.98)
Lao People's Democratic Republic	29.83 (0-154.53)	31.09 (0-158.17)	0.04	1.44 (0-7.51)	0.71 (0-3.57)	-2.49 (-2.57--2.41)
Latvia	61.9 (0-335.41)	30.4 (0-160.42)	-0.51	1.73 (0-9.34)	0.78 (0-4.05)	-2.7 (-2.84--2.56)
Lebanon	11.35 (0-67.78)	16.31 (0-98.14)	0.44	0.54 (0-3.23)	0.27 (0-1.59)	-1.95 (-2.12--1.79)
Lesotho	5.24 (0-29.4)	8.71 (0-49.6)	0.66	0.64 (0-3.64)	0.81 (0-4.67)	1.49 (1.07-1.92)
Liberia	8.96 (0-48.68)	14.33 (0-78.88)	0.6	0.83 (0-4.48)	0.75 (0-4.11)	-0.23 (-0.38--0.07)
Libya	7.67 (0-46.84)	17.23 (0-106.79)	1.25	0.41 (0-2.55)	0.33 (0-2.06)	-0.37 (-0.54--0.2)
Lithuania	82.89 (0-435.34)	46.77 (0-240.8)	-0.44	1.84 (0-9.65)	0.81 (0-4.17)	-2.58 (-2.7--2.46)

Luxembourg	5.38 (0-27.86)	3.75 (0-19.77)	-0.3	0.99 (0-5.15)	0.33 (0-1.74)	-3.41 (-3.51- -3.31)
Macedonia	32.96 (0-157.63)	36.23 (0-177.78)	0.1	1.83 (0-8.79)	1.17 (0-5.72)	-1.76 (-2.01- -1.5)
Madagascar	36.08 (0-193.99)	53.49 (0-277.18)	0.48	0.73 (0-3.9)	0.51 (0-2.6)	-1.26 (-1.32- -1.2)
Malawi	12.17 (0-60.76)	17.85 (0-91.51)	0.47	0.33 (0-1.66)	0.25 (0-1.29)	-1.19 (-1.44- -0.94)
Malaysia	66.37 (0-329.25)	141.02 (0-713.21)	1.12	0.74 (0-3.66)	0.53 (0-2.67)	-1.29 (-1.43- -1.16)
Maldives	0.68 (0-3.47)	0.73 (0-3.77)	0.07	0.8 (0-4.05)	0.23 (0-1.18)	-4.37 (-4.59- -4.14)
Mali	61.44 (0-329.34)	103.87 (0-545.61)	0.69	1.62 (0-8.69)	1.25 (0-6.52)	-0.59 (-0.7-- 0.47)
Malta	4.36 (0-22.32)	3.75 (0-19.29)	-0.14	1.04 (0-5.3)	0.37 (0-1.87)	-3.18 (-3.33- -3.03)
Marshall Islands	0.28 (0-1.44)	0.44 (0-2.33)	0.57	1.82 (0-9.37)	1.4 (0-7.27)	-0.76 (-0.84- -0.67)
Mauritania	8.52 (0-45.77)	13.17 (0-71.41)	0.55	0.9 (0-4.84)	0.67 (0-3.58)	-0.82 (-1.08- -0.56)
Mauritius	9.3 (0-46.77)	14.79 (0-72.96)	0.59	1.33 (0-6.68)	0.82 (0-4.08)	-2.31 (-2.68- -1.95)
Mexico	495.67 (0-2517.86)	877.35 (0-4572.92)	0.77	1.29 (0-6.54)	0.71 (0-3.7)	-2.08 (-2.22- -1.94)
Micronesia (Federated States of)	0.83 (0-4.33)	0.9 (0-4.67)	0.08	1.79 (0-9.35)	1.35 (0-6.9)	-0.94 (-1.03- -0.85)
Moldova	62.21 (0-334.84)	34.59 (0-187.71)	-0.44	1.41 (0-7.6)	0.58 (0-3.15)	-2.32 (-2.67- -1.97)
Monaco	0.66 (0-3.47)	0.55 (0-2.96)	-0.17	0.89 (0-4.66)	0.53 (0-2.81)	-1.65 (-1.78-

						-1.53)
Mongolia	46.68 (0-230.18)	65.24 (0-339.67)	0.4	4.52 (0-22.13)	2.96 (0-15.68)	-1.77 (-1.92- -1.62)
Montenegro	4.44 (0-21.55)	6.23 (0-31.31)	0.4	0.72 (0-3.51)	0.66 (0-3.28)	-0.43 (-0.62- -0.24)
Morocco	24.09 (0-138.47)	42.99 (0-263.23)	0.78	0.17 (0-0.98)	0.13 (0-0.78)	-0.83 (-0.91- -0.75)
Mozambique	36.39 (0-176.78)	60.84 (0-319.44)	0.67	0.7 (0-3.42)	0.64 (0-3.34)	0.14 (-0.02- 0.3)
Myanmar	300.61 (0-1562.09)	284.72 (0-1417.16)	-0.05	1.31 (0-6.84)	0.61 (0-3.02)	-2.83 (-2.96- -2.69)
Namibia	1.5 (0-8.17)	2.41 (0-13.16)	0.61	0.24 (0-1.28)	0.18 (0-0.97)	-1.15 (-1.45- -0.84)
Nauru	0.09 (0-0.45)	0.09 (0-0.46)	0	2.07 (0-10.65)	1.64 (0-8.29)	-0.84 (-1.08- -0.59)
Nepal	61.23 (0-322.29)	110.06 (0-558.04)	0.8	0.66 (0-3.49)	0.49 (0-2.5)	-0.71 (-0.98- -0.44)
Netherlands	181.37 (0-975.81)	125.7 (0-672.7)	-0.31	0.89 (0-4.78)	0.34 (0-1.81)	-3.12 (-3.23- -3.01)
New Zealand	27.39 (0-143.13)	29.02 (0-157.23)	0.06	0.7 (0-3.65)	0.34 (0-1.84)	-2.37 (-2.63- -2.11)
Nicaragua	16.76 (0-84.91)	37.35 (0-189.36)	1.23	1.14 (0-5.74)	0.79 (0-3.99)	-1.1 (-1.37- -0.83)
Niger	24.84 (0-133.03)	61.92 (0-338.79)	1.49	0.97 (0-5.22)	0.85 (0-4.69)	-0.09 (-0.26- 0.08)
Nigeria	107.97 (0-583.78)	149.55 (0-825.15)	0.39	0.26 (0-1.4)	0.18 (0-0.97)	-1.07 (-1.19- -0.95)
Niue	0.03 (0-0.13)	0.02 (0-0.1)	-0.33	1.16 (0-5.68)	0.92 (0-4.57)	-0.92 (-0.97- -0.87)
Northern	0.2	0.48	1.4	1.38 (0-7.05)	1.1 (0-5.55)	-0.68

Mariana Islands	(0-1.06)	(0-2.39)				(-0.95--0.42)
Norway	54.72 (0-291.5)	28.07 (0-150.89)	-0.49	0.77 (0-4.09)	0.26 (0-1.41)	-3.49 (-3.55--3.43)
Oman	4.16 (0-23.94)	5.18 (0-30.53)	0.25	0.61 (0-3.55)	0.28 (0-1.66)	-2.15 (-2.29--2.01)
Pakistan	223.15 (0-1162.32)	448.12 (0-2197.83)	1.01	0.41 (0-2.15)	0.38 (0-1.89)	-0.44 (-0.71--0.17)
Palau	0.17 (0-0.92)	0.29 (0-1.48)	0.71	1.97 (0-10.21)	1.49 (0-7.57)	-0.76 (-0.83--0.68)
Palestine	5.07 (0-31.51)	7.43 (0-43.86)	0.47	0.61 (0-3.82)	0.31 (0-1.87)	-2.23 (-2.47--1.98)
Panama	16.82 (0-84.37)	36.15 (0-192.12)	1.15	1.17 (0-5.86)	0.81 (0-4.31)	-1.14 (-1.29--0.99)
Papua New Guinea	23.12 (0-126.73)	50.79 (0-276.4)	1.2	1.44 (0-7.6)	1.13 (0-5.96)	-0.79 (-0.84--0.75)
Paraguay	18.21 (0-92.54)	39.63 (0-204.95)	1.18	0.85 (0-4.33)	0.7 (0-3.63)	-0.85 (-1.08--0.63)
Peru	269.39 (0-1383.11)	531.44 (0-2774.1)	0.97	2.33 (0-12.03)	1.6 (0-8.33)	-1.56 (-1.83--1.3)
Philippines	131.54 (0-662.82)	289.05 (0-1485.59)	1.2	0.47 (0-2.34)	0.36 (0-1.86)	-0.64 (-0.72--0.55)
Poland	731.6 (0-3648.95)	507.23 (0-2513)	-0.31	1.69 (0-8.4)	0.7 (0-3.46)	-2.99 (-3.12--2.87)
Portugal	265.05 (0-1461.02)	197.39 (0-1062.49)	-0.26	1.97 (0-10.86)	0.78 (0-4.15)	-2.88 (-2.93--2.84)
Puerto Rico	33.15 (0-169.99)	24.2 (0-126.29)	-0.27	0.93 (0-4.77)	0.32 (0-1.69)	-3.7 (-3.84--3.56)
Qatar	0.78 (0-4.45)	2.29 (0-13.25)	1.94	0.85 (0-4.92)	0.29 (0-1.75)	-3.69 (-4.32--3.06)

Romania	348.09 (0-1794.63)	322.72 (0-1612.27)	-0.07	1.26 (0-6.47)	0.87 (0-4.33)	-1.4 (-1.55- -1.24)
Russian Federation	4499.25 (0-23008.44)	2438.74 (0-12348.06)	-0.46	2.48 (0-12.65)	1.02 (0-5.15)	-3.05 (-3.16- -2.94)
Rwanda	30.81 (0-156.62)	31.66 (0-164.25)	0.03	1.11 (0-5.57)	0.55 (0-2.81)	-3.32 (-3.69- -2.94)
Saint Kitts and Nevis	0.49 (0-2.5)	0.43 (0-2.37)	-0.12	1.31 (0-6.72)	0.69 (0-3.84)	-1.77 (-1.95- -1.59)
Saint Lucia	1.37 (0-7.18)	1.94 (0-10.74)	0.42	1.69 (0-8.8)	0.82 (0-4.55)	-2.8 (-3.09- -2.51)
Saint Vincent and the Grenadines	0.95 (0-4.9)	1.09 (0-5.79)	0.15	1.37 (0-7.07)	0.79 (0-4.21)	-1.76 (-1.99- -1.53)
Samoa	0.56 (0-3.36)	0.85 (0-4.98)	0.52	0.75 (0-4.36)	0.65 (0-3.77)	-0.54 (-0.63- -0.45)
San Marino	0.73 (0-3.79)	0.6 (0-3.27)	-0.18	2.02 (0-10.43)	0.71 (0-3.94)	-2.54 (-2.86- -2.22)
Sao Tome and Principe	0.74 (0-3.9)	1.09 (0-5.69)	0.47	1.23 (0-6.42)	1.12 (0-5.84)	-0.22 (-0.37- -0.07)
Saudi Arabia	19.97 (0-120.87)	37.05 (0-211.98)	0.86	0.35 (0-2.11)	0.19 (0-1.11)	-2.1 (-2.28- -1.91)
Senegal	27.63 (0-148.91)	55.86 (0-305.9)	1.02	0.91 (0-4.85)	0.78 (0-4.27)	-0.18 (-0.41- 0.05)
Serbia	118.39 (0-587.02)	108.61 (0-534.05)	-0.08	1.16 (0-5.75)	0.65 (0-3.18)	-2.27 (-2.48- -2.06)
Seychelles	0.49 (0-2.45)	0.54 (0-2.71)	0.1	0.87 (0-4.36)	0.48 (0-2.42)	-1.78 (-1.92- -1.64)
Sierra Leone	16.37 (0-89.39)	27 (0-148.77)	0.65	0.84 (0-4.56)	0.77 (0-4.24)	0.18 (-0.01- 0.37)
Singapore	28.86 (0-141.15)	31.16 (0-154.89)	0.08	1.37 (0-6.71)	0.38 (0-1.87)	-4.27 (-4.51-

						-4.03)
Slovakia	90.12 (0-438.73)	65.56 (0-330.1)	-0.27	1.5 (0-7.32)	0.68 (0-3.44)	-2.49 (-2.59--2.39)
Slovenia	39.69 (0-195.52)	26.73 (0-132.21)	-0.33	1.6 (0-7.88)	0.56 (0-2.81)	-3.5 (-3.61--3.39)
Solomon Islands	2.37 (0-13.31)	4.94 (0-26.25)	1.08	1.92 (0-10.34)	1.52 (0-7.89)	-0.71 (-0.82--0.6)
Somalia	28.44 (0-143.94)	55.75 (0-288.99)	0.96	1.2 (0-5.98)	0.94 (0-4.85)	-0.89 (-0.96--0.81)
South Africa	102.46 (0-567.57)	166.13 (0-930.99)	0.62	0.49 (0-2.72)	0.37 (0-2.09)	-1.14 (-1.45--0.83)
South Korea	1348.47 (0-6571.07)	1028.66 (0-5083.89)	-0.24	4.61 (0-22.14)	1.11 (0-5.5)	-4.99 (-5.14--4.84)
South Sudan	22.41 (0-116.02)	27.4 (0-144.25)	0.22	0.9 (0-4.63)	0.75 (0-3.94)	-0.81 (-0.97--0.64)
Spain	499.56 (0-2918.79)	354.36 (0-2055.04)	-0.29	0.93 (0-5.37)	0.36 (0-2.03)	-2.88 (-3.01--2.75)
Sri Lanka	73.86 (0-360.4)	77.86 (0-406.55)	0.05	0.73 (0-3.59)	0.29 (0-1.54)	-3.03 (-3.24--2.82)
Sudan	103.56 (0-622)	150.23 (0-953.14)	0.45	1.1 (0-6.66)	0.78 (0-5)	-1.16 (-1.22--1.11)
Suriname	2.07 (0-11.03)	3.23 (0-17.89)	0.56	0.85 (0-4.5)	0.52 (0-2.87)	-1.45 (-1.66--1.23)
Swaziland	2.13 (0-12.08)	3.47 (0-19.86)	0.63	0.77 (0-4.28)	0.62 (0-3.49)	-0.27 (-0.82-0.28)
Sweden	111.05 (0-587.48)	60.02 (0-314.08)	-0.46	0.7 (0-3.69)	0.25 (0-1.32)	-3.44 (-3.64--3.25)
Switzerland	84.96 (0-450.76)	54.93 (0-291.46)	-0.35	0.79 (0-4.16)	0.28 (0-1.49)	-3 (-3.35--2.65)
Syria	21.67	37.4	0.73	0.42 (0-2.55)	0.31 (0-1.81)	-1.27

	(0-129.63)	(0-221.19)				(-1.41--1.13)
Taiwan (Province of China)	240.76 (0-1210.79)	309.97 (0-1568.56)	0.29	1.59 (0-7.97)	0.72 (0-3.66)	-3 (-3.22--2.77)
Tajikistan	63.24 (0-316.57)	62.38 (0-333.05)	-0.01	2.32 (0-11.6)	1.12 (0-6)	-2.24 (-2.49--1.99)
Tanzania	80.82 (0-398.45)	122.22 (0-616.37)	0.51	0.78 (0-3.8)	0.5 (0-2.53)	-1.6 (-1.68--1.51)
Thailand	327.99 (0-1636.8)	637.54 (0-3388.31)	0.94	0.95 (0-4.72)	0.6 (0-3.18)	-1.87 (-2.02--1.72)
The Bahamas	1.57 (0-8.28)	2.55 (0-14.05)	0.62	1.04 (0-5.44)	0.66 (0-3.61)	-1.44 (-1.57--1.3)
The Gambia	1.01 (0-5.55)	2.4 (0-12.76)	1.38	0.3 (0-1.64)	0.26 (0-1.36)	-0.54 (-0.67--0.41)
Timor-Leste	2.38 (0-12.44)	5.23 (0-26.86)	1.2	0.88 (0-4.64)	0.64 (0-3.31)	-0.93 (-1.16--0.69)
Togo	10.12 (0-54.41)	29.9 (0-170.41)	1.95	0.88 (0-4.73)	0.86 (0-4.82)	0.26 (0.1-0.41)
Tokelau	0.02 (0-0.1)	0.01 (0-0.06)	-0.5	1.39 (0-7.41)	0.86 (0-4.39)	-1.66 (-1.69--1.64)
Tonga	0.83 (0-4.39)	1.03 (0-5.27)	0.24	1.65 (0-8.68)	1.33 (0-6.72)	-0.62 (-0.74--0.5)
Trinidad and Tobago	6.91 (0-36.22)	7.36 (0-38.5)	0.07	0.87 (0-4.53)	0.39 (0-2.02)	-2.78 (-2.99--2.57)
Tunisia	16.73 (0-98.76)	28.15 (0-168.08)	0.68	0.34 (0-2.03)	0.21 (0-1.28)	-1.7 (-1.78--1.61)
Turkey	385.5 (0-2389.32)	470.35 (0-2934.62)	0.22	1.11 (0-6.89)	0.51 (0-3.18)	-2.65 (-2.99--2.31)
Turkmenistan	33.88 (0-167.8)	30.23 (0-159.07)	-0.11	1.78 (0-8.68)	0.74 (0-3.9)	-2.85 (-3.13--2.56)

Tuvalu	0.11 (0-0.59)	0.11 (0-0.59)	0	1.77 (0-9.49)	1.18 (0-6.12)	-1.22 (-1.29--1.15)
Uganda	45.83 (0-233.84)	68.97 (0-355.62)	0.5	0.75 (0-3.82)	0.5 (0-2.58)	-1.94 (-2.23--1.65)
Ukraine	1306.35 (0-7142.17)	541.02 (0-3006.87)	-0.59	1.83 (0-9.99)	0.71 (0-3.96)	-3.49 (-3.67--3.3)
United Arab Emirates	3.37 (0-19.94)	11.16 (0-66.59)	2.31	0.77 (0-4.73)	0.39 (0-2.41)	-0.8 (-1.32--0.29)
United Kingdom	774.64 (0-4185.52)	448.62 (0-2434.7)	-0.42	0.81 (0-4.4)	0.32 (0-1.71)	-3.04 (-3.15--2.92)
United States	1217.15 (0-6444.34)	1255.31 (0-6523.05)	0.03	0.38 (0-2)	0.22 (0-1.13)	-1.89 (-1.95--1.83)
Uruguay	52.74 (0-264.21)	50.05 (0-253.48)	-0.05	1.35 (0-6.75)	0.87 (0-4.42)	-1.53 (-1.64--1.42)
Uzbekistan	192.97 (0-972.54)	157.63 (0-805.41)	-0.18	1.67 (0-8.4)	0.59 (0-3.03)	-3.01 (-3.2--2.82)
Vanuatu	0.91 (0-4.95)	2.04 (0-10.78)	1.24	1.62 (0-8.46)	1.29 (0-6.63)	-0.87 (-0.92--0.82)
Venezuela	150.87 (0-756.02)	256.4 (0-1313.88)	0.7	1.64 (0-8.26)	0.88 (0-4.5)	-2.46 (-2.65--2.26)
Viet Nam	490.47 (0-2475.18)	630.08 (0-3105.65)	0.28	1.23 (0-6.16)	0.64 (0-3.15)	-2.49 (-2.69--2.29)
Virgin Islands, U.S.	0.7 (0-3.68)	0.74 (0-3.83)	0.06	0.91 (0-4.64)	0.42 (0-2.15)	-2.43 (-2.61--2.24)
Yemen	65.1 (0-410.83)	136.27 (0-890.04)	1.09	1.3 (0-8.24)	0.98 (0-6.44)	-1.14 (-1.23--1.04)
Zambia	23.51 (0-121.51)	38.85 (0-209.29)	0.65	0.86 (0-4.45)	0.59 (0-3.1)	-1.68 (-1.94--1.42)
Zimbabwe	33.02 (0-174.9)	68.89 (0-371.76)	1.09	0.88 (0-4.65)	1.02 (0-5.37)	1.11 (0.62-1

.59)

Supplementary table 2. The deaths of GC-DHIS cases and rates in 1990 and 2021 across 204 countries, and the trends from 1990 to 2021.
