# Supplementary material for: Global, regional, and national burden of gastric cancer attributable to diet high in sodium from 1990 to 2021
Source: Front Nutr. 2025 Oct 7;12:1674979. doi: 10.3389/fnut.2025.1674979 (PMC12537357; doi:10.3389/fnut.2025.1674979)
Supplement: Supplementary file 2 [file Data_Sheet_2.PDF]

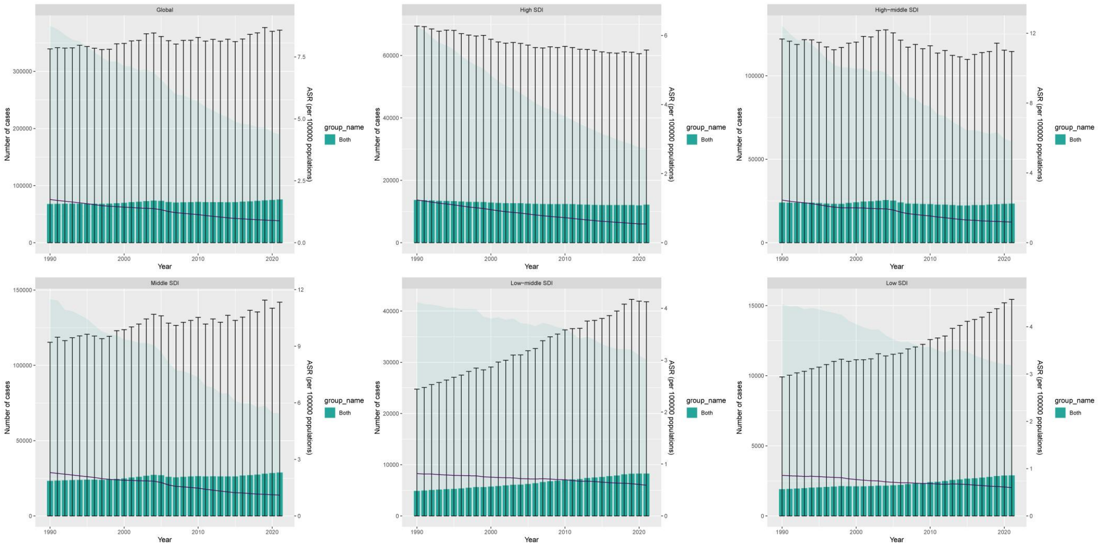

Supplementary figure 1. Death cases and ASMR of GC-DHIS from 1990 to 2021.

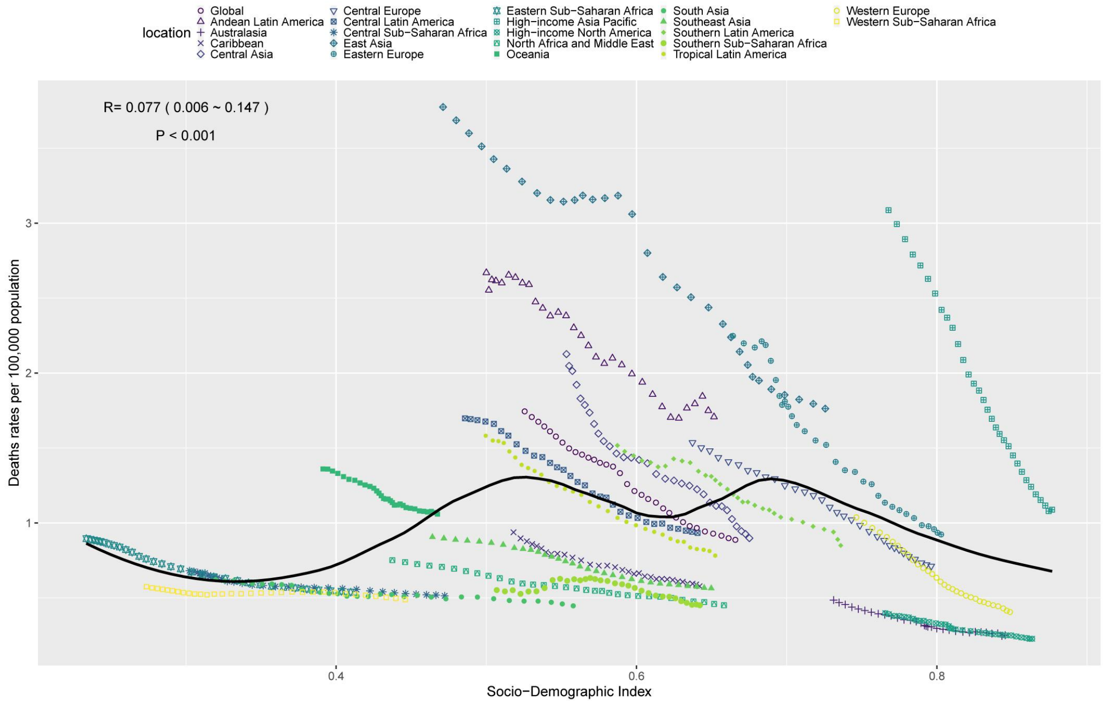

Supplementary figure 2. ASMR of GC-DHIS in 21 GBD regions by SDI, 1990–2021.
